# Control of recollection by slow gamma dominating mid-frequency gamma in hippocampus CA1

**DOI:** 10.1371/journal.pbio.2003354

**Published:** 2018-01-18

**Authors:** Dino Dvorak, Basma Radwan, Fraser T. Sparks, Zoe Nicole Talbot, André A. Fenton

**Affiliations:** 1 Center for Neural Science, New York University, New York, New York, United States of America; 2 School of Medicine, New York University, New York, New York, United States of America; 3 Neuroscience Institute at the New York University Langone Medical Center, New York, New York, United States of America; 4 Department of Physiology and Pharmacology, The Robert F. Furchgott Center for Neural & Behavioral Science, State University of New York, Downstate Medical Center, Brooklyn, New York, United States of America; Institute of Science and Technology Austria, Austria

## Abstract

Behavior is used to assess memory and cognitive deficits in animals like Fmr1-null mice that model Fragile X Syndrome, but behavior is a proxy for unknown neural events that define cognitive variables like recollection. We identified an electrophysiological signature of recollection in mouse dorsal Cornu Ammonis 1 (CA1) hippocampus. During a shocked-place avoidance task, slow gamma (SG) (30–50 Hz) dominates mid-frequency gamma (MG) (70–90 Hz) oscillations 2–3 s before successful avoidance, but not failures. Wild-type (WT) but not Fmr1-null mice rapidly adapt to relocating the shock; concurrently, SG/MG maxima (SG_dom_) decrease in WT but not in cognitively inflexible Fmr1-null mice. During SG_dom_, putative pyramidal cell ensembles represent distant locations; during place avoidance, these are avoided places. During shock relocation, WT ensembles represent distant locations near the currently correct shock zone, but Fmr1-null ensembles represent the formerly correct zone. These findings indicate that recollection occurs when CA1 SG dominates MG and that accurate recollection of inappropriate memories explains Fmr1-null cognitive inflexibility.

## Author summary

Behavior is often used as proxy to study memory and cognitive deficits in animals like Fmr1-KO mice that model Fragile X Syndrome, the most prevalent single-gene cause of intellectual disability and autism. However, it is unclear what neural events define cognitive variables like recollection of memory and cognitive inflexibility. We identified a signature of recollection in the local field potentials of mouse dorsal CA1 hippocampus. When mice on a rotating platform avoided an invisible, fixed shock zone, slow gamma (30–50 Hz) oscillations dominated mid-frequency gamma (70–90 Hz) oscillations (SG_dom_) 2–3 s before mice successfully avoided the shock zone. Wild-type but not Fmr1-KO mice adapt to relocating the shock zone; concurrently, SG_dom_ decreases in wild-type but not in cognitively inflexible Fmr1-KO mice. During SG_dom_, principal cell ensembles represent distant locations; during place avoidance, these are avoided places in the shock zone vicinity. During shock relocation, wild-type ensembles encode distant locations near the currently correct shock zone, but Fmr1-KO ensembles manifest representational inflexibility, encoding the formerly correct zone. These findings suggest evidence for competition amongst CA1 inputs for CA1 information-processing modes and indicate that recollection occurs when CA1 slow gamma dominates mid-frequency gamma and that accurate recollection of inappropriate memories explains Fmr1-KO cognitive inflexibility.

## Introduction

The hippocampus is crucial for both learning and remembering information, especially about space [1], and because the same place-representing neurons participate in both processes [2–7], it is unknown what neural events control whether hippocampal neurons are encoding current experience or recollecting information from memory [8]. A prominent “communication-through-coherence” [9–12] or “routing-by-synchrony” hypothesis asserts that activity in Cornu Ammonis 1 (CA1) switches between an information-acquiring mode associated with mid-frequency gamma (MG) (60–90-Hz) oscillations that synchronize hippocampus output with neocortical input and a separate, long-term memory–recollection mode associated with slow gamma (SG) (30–60-Hz) oscillations that synchronize CA1 output with intrahippocampal Cornu Ammonis 3 (CA3)→CA1 inputs [12,13]. Gamma oscillations are generated by local interneurons [14–17], and the local CA1 GABAergic currents that underlie gamma oscillations are effectively driven by tonic excitation, as described by pyramidal interneuron network gamma (PING) models of gamma generation [18–20]. Furthermore, tonic inputs to PING as well as interneuron network gamma (ING) models can locally generate distinct lower and higher frequency gamma oscillations by local competition between distinct interneuron populations with correspondingly long- and short-lasting postsynaptic inhibition [21]. Because CA1 receives two anatomically distinct inputs [22,23] and each mediates both dendritic excitation and feed-forward inhibition [24], routing-by-synchrony hypotheses predict that during long-term memory recall, the CA3-associated SG input will outcompete the entorhinal cortex-associated MG input for control of CA1 output.

We test this prediction and find in freely behaving mice solving a place task that SG and MG oscillations are concurrent in mouse CA1, but a transient dominance of SG oscillations over MG oscillations signals recollection. This SG dominance lasts several hundred milliseconds and occurs on average approximately every 9 s, both when mice are active and still. Increased and decreased rates of SG dominance predict accurate, failed, and changed place memory in wild-type (WT) mice as well as cognitive inflexibility in a Fmr1-null mutant mouse model of Fragile X Syndrome (FXS) intellectual disability, which is associated with high prevalence of autism. During SG dominance, putative pyramidal cell ensemble discharge represents distant locations, and during place avoidance tasks, these distant locations are the vicinity of the shock zone that the mouse learned to avoid. However, when Fmr1-null mice express cognitive inflexibility by continuing to avoid the formerly correct and now incorrect place, these SG dominance events are excessive and predictive of putative pyramidal cell representations of formerly correct shock-zone location memories. Because gamma oscillations are generated by local inhibitory synapses, and consistent with theory [21], these results point to local biases in competing gamma-generating inhibitory events as the potential origin of distinct information and long-term memory–processing modes, such as recollection.

## Results

### Identifying recollection events prior to active avoidance

**Fig 1:**
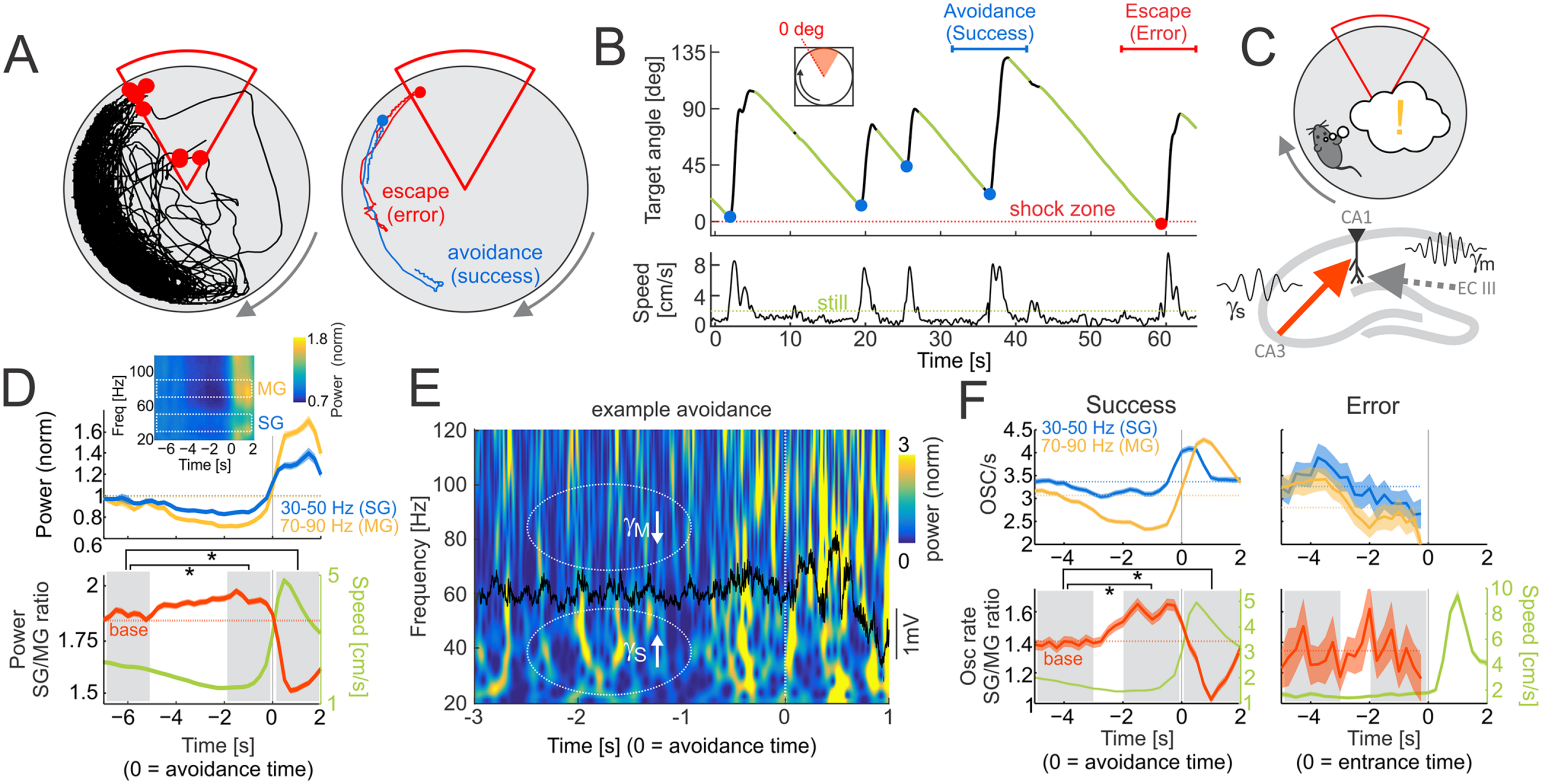
SG dominates MG prior to successful place avoidance. (A) Left: typical 30-min path during the third active place avoidance training session. Shocks are shown as red dots. Right: example of avoidance (success; blue line) and escape after receiving a shock (error; red line). (B) Top: time profile of the angular distance to the leading edge of the shock zone, showing a typical sawtooth avoidance pattern during approximately 60 s. Periods of stillness (green) when the mouse is passively carried towards the shock zone are interrupted by active avoidances (blue dots). Entrance to the shock zone is marked as a red dot. The horizontal blue and red lines mark time intervals of the example avoidance and escape from panel (A), right. The red dotted line marks the leading edge of the shock zone. Bottom: speed profile during the same approximately 60-s interval. The stillness threshold is shown as a green dotted line at 2 cm/s. (C) Schematic depiction of the working hypothesis—as the mouse approaches the shock zone (top), SG driven by CA3 inputs transiently dominates MG driven by ECIII inputs, causing recollection of the shock zone location. (D) Top: average power of SG (blue; 30–50 Hz) and MG (yellow; 70–90 Hz) in the LFP around the time of avoidance initiation (*T* = 0). Mean powers are displayed as dotted lines. Inset shows average of normalized power across 20–120 Hz around avoidance initiation. Representative SG and MG bands are marked by white rectangles. Bottom: the average ratio of SG to MG power (red line) around avoidance initiation. The mean power ratio is shown as a dotted line. The corresponding average speed profile is shown in green. Data are represented as average ± SEM. Gray boxes represent time intervals for statistical comparisons, *p < 0.05 relative to baseline (−7–−5 s). (E) The time-frequency representation of a 4-s example LFP (overlaid in black) around the initiation of an avoidance start (*T* = 0 marks avoidance initiation). Notice the relative reduction in number of MG (70–90 Hz) oscillatory events relative to SG (30–50 Hz) events prior to the avoidance (*T* = approximately −2 s) compared to times during the active avoidance (*T* > 0 s). (F) Left, top: average event rates for SG (blue; 30–50 Hz) and MG (yellow; 70–90 Hz) oscillations around the time of avoidance initiation (*T* = 0). Mean rates are displayed as dotted lines. Left, bottom: the average ratio of SG to MG event rates (red line) around avoidance initiation. The mean ratio is shown as a dotted line. The corresponding average speed profile is shown in green. Right: same as (F), left, but for avoidance errors. Data are represented as average ± SEM. Gray boxes represent time intervals for statistical comparisons, *p < 0.05 relative to baseline (−5–−3 s). CA3, Cornu Ammonis 3; ECIII, entorhinal cortex layer 3; LFP, local field potential; MG, mid-frequency gamma; SG, slow gamma. *Underlying data can be found here*: [https://goo.gl/oHH22A].

We began by identifying when mice were likely to recall the location of shock during training in variants of the active place avoidance task (Fig 1A) [25]. Periods of stillness when the mouse is passively carried towards the shock zone are interrupted by active avoidances (Fig 1B), indicating successful recollection of the shock location and identifying times with a high likelihood of recollection (Fig 1).

The routing-by-synchrony hypothesis [12,13] predicts that CA3-driven SG oscillations will transiently dominate neocortex-driven MG oscillations when the mouse is recollecting the shock zone location (Fig 1C). Concurrent local field potentials (LFPs) reflecting synchronous synaptic activity within the dorsal hippocampus were recorded at the perisomatic region of CA1 and examined during these behavioral segments with a high likelihood of recollection. The LFP state was mostly in theta, although of somewhat lower amplitude during stillness (S1A Fig), as is typical for spatially alert stillness [26]. At stratum pyramidale, SG and MG power could be separated by their different phase relationships to theta but less so by their frequency content during both stillness and active locomotion (S2C and S2D Fig). Importantly, the rate of sharp-wave ripples (SWRs) during these pre-avoidance periods of stillness was no different than the overall stillness ripple rate (S1C Fig).

It was reported that theta oscillations in the stratum pyramidale LFP of the freely-behaving rat are predominantly concurrent with either 25–50-Hz CA3-associated gamma or 65–140-Hz entorhinal cortex layer 3 (ECIII) gamma oscillations, but rarely both, and the slower gamma tends to occur at an earlier theta phase than the faster gamma [12]. In contrast to those recordings in the rat, we find that, in the mouse, both SG and MG oscillations frequently occur within single theta oscillations in the stratum pyramidale LFP (S2E and S3D Figs). It is only after selecting oscillations with the largest power that single theta cycles can be shown to be dominated by either SG or MG oscillations, but this is likely an artifact of rejecting most oscillations, because only a single supra-threshold gamma oscillation occurs within a single theta cycle when the threshold is >2 SD (S3D Fig). Furthermore, we also find in the mouse that SG oscillations occur close to the theta trough, while MG oscillations occur close to the theta peak (S2C and S2D Fig). This is opposite to the relationship reported by Colgin et al. 2009 but is similar to what is reported by other work in rats [14] and mouse [16,27]. While input-specific oscillatory components in CA1 can be demixed using high-density silicon probe recordings with current source density (CSD) analysis [27] (S2B and S2C Fig) or independent component analysis [28], here we exploit that both SG and MG oscillations can be identified in CA1 stratum pyramidale, which is both the target of place cell recordings and the basis of virtually all the data upon which the routing-by-synchrony hypotheses are based.

We began by comparing power in representative frequency bands for SG (30–50 Hz) and MG (70–90 Hz) and their respective power ratio (Fig 1D). Before the mouse initiated successful avoidance movements, the ratio of SG to MG power progressively increased from about 5 s prior to the initiation of avoidance movements, with the maximum ratio occurring about 1 s prior to the active avoidance. This relationship was confirmed with one-way ANOVA (F_2,5168_ = 294.84; *p* = 5.6 × 10^−122^) of the differences between three time intervals (−7–−5 s, −2–0 s, and 0–2 s) around the avoidance onset. Post hoc Dunnett’s tests confirmed significant differences from the −7–−5-s baseline interval for intervals just before (−2–0 s) and just after (0–2 s) the initiation of active avoidance (*p* < 0.001 in both cases). The power ratio was strongly negatively correlated with speed (Fig 1D, bottom; Pearson’s correlation *r* = −0.25, *p* = 0), as has been reported [29]. Because changes in speed confound associating these changes in the LFP with recollection, we examined alternative approaches for characterizing gamma changes in the LFP that are minimally impacted by speed and instead emphasized the internal cognitive information processing upon which the routing-by-synchrony hypothesis is based.

The routing-by-synchrony hypothesis also predicts that information between two networks is relayed most effectively during high-power, synchronized oscillatory states, in contrast to all non-oscillatory activity, which gives rise to the 1/f power spectra of LFP and electroencephalogram (EEG) signals [30] (S1B Fig). Because the present work relies on comparing oscillations of different frequency bands, to avoid potentially misleading estimates of the relative strength of oscillations from 1/f organized power spectra, we built on our prior work and discretized continuous LFP signals into frequency-specific oscillatory events and their rates [31]. Oscillatory events were detected as local power maxima in the z-score normalized, wavelet-transformed LFP signal (S2E Fig; also refer to S3 Fig for discussion about threshold setting for event detection). To compute rates of oscillatory events, we first selected representative frequency bands for SG (30–50 Hz) and MG events (70–90 Hz; refer to S3 Fig for discussion about band selection) and then computed event rates as the number of detected events in a given frequency range above 2.5 SD power at stratum pyramidale in 1,000-ms-long windows advanced by 250 ms, consistent with prior routing-by-synchrony studies [7,12,32]. From now on, we therefore use SG (30–50 Hz) and MG (70–90 Hz) event rates. These are defined as the number of detected oscillatory events in a 1-s-long interval that averages several theta cycles and avoids potential controversies around which oscillations dominate a single theta cycle. We compute their respective ratios: SG/MG as the ratio of the SG to MG event rates and MG/SG as the ratio of MG to SG event rates.

### The ratio of SG to MG oscillation event rates is maximal when recollection likelihood is high

The session-specific SG and MG oscillation rates and the SG/MG ratio were examined around the time of successful avoidances of the initial location of shock. The MG oscillation rate decreased, with the minimum occurring 2–0.5 s before avoidance onset (Fig 1F, left; compare to wavelet spectrum in Fig 1E). SG had a less pronounced decrease and could even increase before avoidance onset. SG increased after avoidance onset, peaking about 500 ms afterwards, preceding MG, which peaked at 750 ms. In contrast to the power ratio (Fig 1D), the SG/MG ratio was only weakly correlated with speed (SG/MG ratio: *r* = −0.09, *p* = 4.6 × 10^−22^, explaining <1% of the variance; power ratio: *r* = −0.25, *p* = 0, explaining >6% of the variance; *t* test for difference between means of Fisher-transformed correlations: t_16_ = 2.13, *p* = 0.048). The SG/MG ratio was maximal 1–2 s before and it was minimal about 1 s after avoidance onset (Fig 1F, left). These relationships were confirmed with one-way ANOVA (F_2,4134_ = 54.22; *p* = 5.7 × 10^−24^) on the SG/MG ratios between three time intervals (−5–−3 s, −2–0 s, and 0–2 s) around avoidance onset. Post hoc Dunnett’s test confirmed significant differences from the −5–−3-s baseline interval for intervals just before (−2–0 s) and just after (0–2 s) the initiation of active avoidance (*p* < 0.001 in both cases). The comparison of the two intervals before entering the shock zone was not significant (F_1,79_ = 0.38; *p* = 0.54) when the mouse failed to actively avoid shock but nonetheless initiated running away from the shock zone upon being shocked (Fig 1F, right). Because this SG dominance over MG (SG_dom_) was identified during a few seconds of stillness prior to avoidance of the shock location, it is possible that SG_dom_ either indicates momentary recollection of the shock locations or preparation (initiation) of locomotion.

### SG dominance predicts successful place avoidance

**Fig 2:**
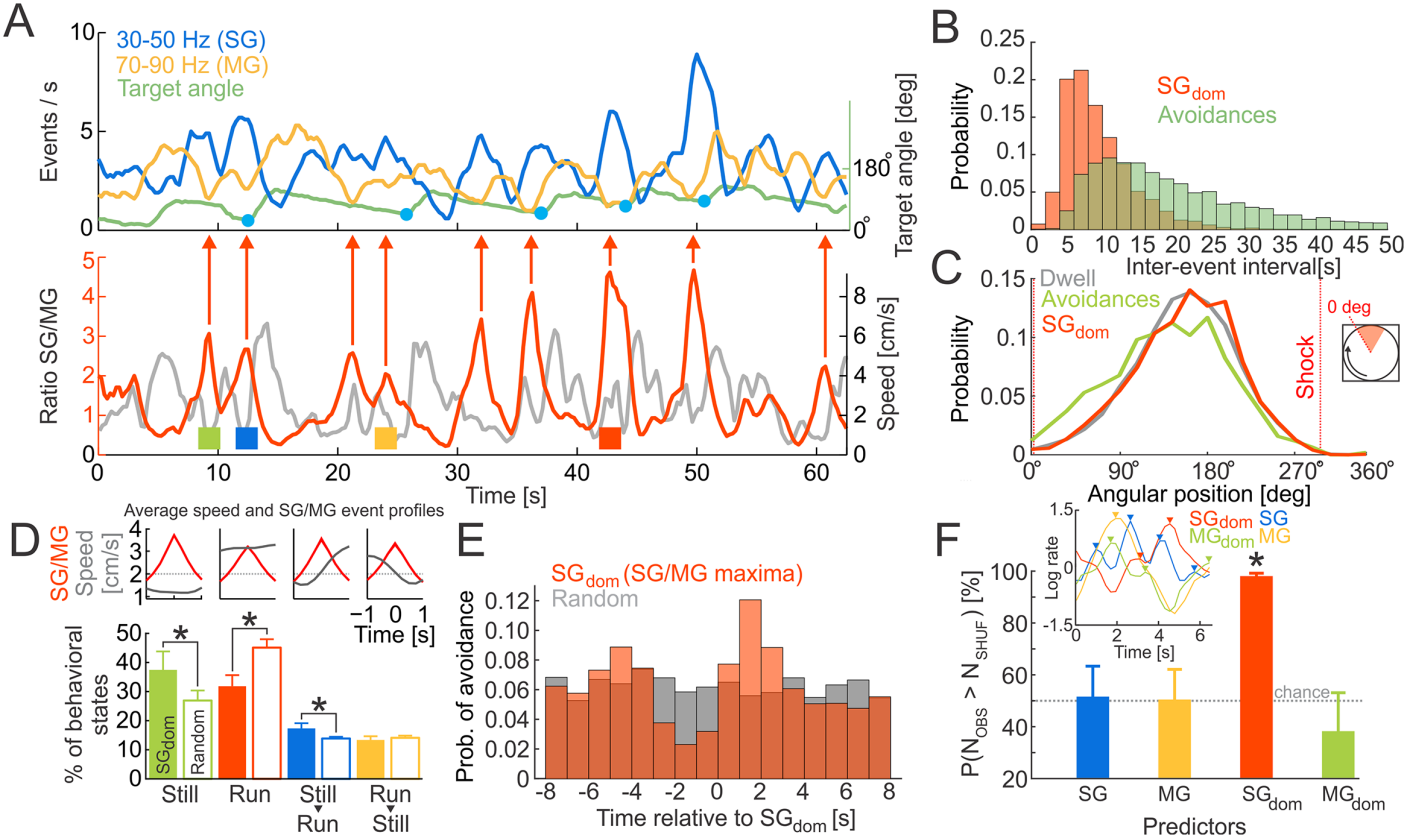
SG dominance predicts active avoidance. (A) Top: time series of SG and MG event rates and the angular distance of a mouse from the leading edge of the shock zone. Avoidances are marked by blue dots. The leading edge of the shock zone corresponds to 0°. Bottom: time series of SG to MG ratio (SG/MG) with local maxima (SG_dom_) indicated (red arrows). (B) Probability distributions of inter-event intervals for consecutive SG_dom_ events and successful avoidances during training sessions. (C) Angular distributions of a mouse’s location (Dwell; gray), locations of avoidances (Avoidances; green), and locations of SG_dom_ events (SG_dom_; red). (D) Proportions of different behavioral events detected during SG_dom_ events (filled bars) and randomly selected events (empty bars). Average SG/MG ratio and speed in 2-s windows centered around SG/MG maxima are shown at the top. Dotted line represents the speed threshold 2 cm/s used for the behavior classification. Corresponding examples of behavioral states are marked by colored squares in (A), bottom. *p < 0.01. (E) Probability of observing an avoidance relative to an SG_dom_ event and randomly selected times. (F) The probability of predicting avoidances by chance (after randomly shifting the time stamps of detected maxima), by using the maxima of the SG or MG, the SG_dom_ events, or the MG/SG ratio maxima (MG_dom_ events). The inset shows examples of detected maxima in the four series types. *p < 0.05 relative to chance. Data are represented as average ± SEM. MG, mid-frequency gamma; N_OBS_, number of observed events; N_SHUF_, number of shuffled events; SG, slow gamma. *Underlying data can be found here*: [https://goo.gl/oHH22A].

We then investigated whether the rates of SG or MG oscillations or their ratio indexed behavior that is potentially associated with recollection or alternatively, indexes initiation of movement, per se. We first examined the time series of the SG/MG ratio but did not restrict the analysis to peri-avoidance episodes with preceding stillness (Fig 2). To compute time intervals between the SG/MG maxima that define SG_dom_, we first detected local peaks in the SG/MG ratio series with amplitude >1 (i.e., SG > MG) and then selected the subset of maxima with prominence (amplitude difference between maxima to the preceding and following minima) >1. This step excluded short intervals resulting in multiple peaks in a sequence (see Fig 2A).

We first investigated whether the occurrence of SG_dom_ events (average inter-event time: 9.3 s) and avoidances (average inter-event time: 26.0 s; Fig 2B) were substantially similar or different (compare upper and lower time series in Fig 2A). SG_dom_ events were more frequent than avoidances (Kolmogorov-Smirnov test D_3433_ = 0.522, *p* = 3.2 × 10^−8^), indicating that not every SG_dom_ event is followed by avoidance behavior. This means that while SG dominance in the CA1 LFP was initially identified by focusing on peri-avoidance episodes defined by stillness changing to locomotion, a parsimonious account for SG dominance is that it is more likely to indicate moments of active recollection than just preparation for initiating movement (S1 Video).

We next analyzed the length of SG_dom_ events by thresholding the SG/MG ratio time series. The SG_dom_ peaks lasted 3.0 ± 2.7 s when the threshold was SG/MG ≥ 1, and they lasted 1.2 ± 1.1 s when the threshold was SG/MG ≥ 2, suggesting that SG dominance likely lasts several theta cycles.

We then assessed the spatial distribution of SG_dom_ events (Fig 2C). Consistent with these being internal, cognitive events, the spatial distribution of the SG_dom_ events resembles the spatial distribution of where the mice visited (maximal dwell opposite the shock zone; Mann-Whitney-Wilcoxon nonparametric test compared to dwell, *U* = 2.8 × 10^9^, *p* = 0.43), and, accordingly, these places differed from the places in which the mice expressed avoidance behavior by initiating movement away from the leading edge of the shock zone (Mann-Whitney-Wilcoxon nonparametric test compared to dwell, *U* = 9.4 × 10^8^, *p* = 0). These data are consistent with SG_dom_ being related to an internal cognitive variable like active recollection, such that recollection might not only be of the locations of shock.

We next studied in what behavioral states SG_dom_ events occur (Fig 2D). We classified behavioral states using each mouse’s average speed during −1–−0.25 s before and 0.25–1 s after SG_dom_ events. The “Run” state had an average speed before and after a SG_dom_ event of ≥2 cm/s. The “Still” state had an average speed before and after a SG_dom_ event of <2 cm/s. The “Still→Run” state had an average speed before a SG_dom_ event of <2 cm/s and ≥2 cm/s after the SG_dom_ event. The “Run→Still” state had an average speed before a SG_dom_ event of ≥2 cm/s and <2 cm/s after the SG_dom_ event.

SG_dom_ events occur during both active movement and stillness. During pretraining recordings, when the mice explored the rotating arena prior to ever experiencing shock, the majority (approximately 75%) of observations during SG_dom_ or random events comprised continuous stillness or running, with greater prevalence of running. The prevalence of these movement-defined states was indistinguishable during SG dominance and randomly selected episodes (S4B Fig). Place avoidance training changed which movement-defined behaviors were expressed during SG dominance. Overall, the continuous stillness and running behaviors still account for about 70% of observations during SG_dom_ events; transitional behaviors from stillness to running or vice versa are less frequent (Fig 2D; F_3,35_ = 8.88, *p* = 0.0002; post hoc Dunnett’s test against Still: Still = Run > Still→Run > Run→Still). We then computed the frequencies of observing these movement-defined behaviors during the same number of random intervals as were identified for SG_dom_ (empty bars in Fig 2D). Overall, the majority (approximately 75%) of observations were during continuous stillness or running, like during SG dominance. However, the SG_dom_ and random event comparisons indicate that stillness and transitions from stillness to running are overrepresented during SG_dom_, while running is underrepresented during SG_dom_ events (χ^2^ test for multiple proportions, χ^2^_3_ = 119.1, *p* = 8.4 × 10^−25^; Still: χ^2^_1_ = 36.4, *p* = 2.4 × 10^−7^; Run: χ^2^_1_ = 62.6, *p* = 8.1 × 10^−13^; Still→Run: χ^2^_1_ = 19.9, *p* = 0.0005; Run→Still: χ^2^_1_ = 0.15, *p* = 0.99). Thus, prior to the place learning task, the prevalence of movement-related behaviors is indistinguishable from chance during SG dominance, but the prevalence of these behaviors deviates from chance to favor behaviors that are associated with a high likelihood of recollecting the location in which a shock was previously experienced. These investigations of the prevalence of SG_dom_ during movement-defined behaviors indicate it is unlikely that SG_dom_ can be fully explained by movement planning or initiation.

To further evaluate the possibility that SG dominance is indicative of long-term memory recollection, we tested the ability of the SG_dom_ events to predict successful avoidances, reasoning that recollecting locations of shock should precede effective avoidance behavior. First, we examined the probability of observing an avoidance at times relative to SG_dom_ events and compared that distribution to the probability of observing an avoidance at times relative to randomly selected events. The distributions were different, and there was an increased occurrence of avoidances 1–2 s after SG_dom_ events (Fig 2E; Kolmogorov-Smirnov test D_2715_ = 0.08, *p* = 1.6 × 10^−8^). Second, we created four avoidance predictors that used either the maxima in SG rate, maxima in MG rate, maxima in SG/MG ratio (i.e., SG_dom_), or maxima in the MG/SG ratio (i.e., MG_dom_). Prior to detecting these peaks, the ratios (SG/MG and MG/SG) were log-transformed and all time series were z-score normalized; only maxima with z-score values >0.5 SD were selected, to guarantee similar rates of detected peaks in all four time series. Avoidances were predicted in a 4-s-long window following the maxima. Note that even though the MG/SG ratio is the inverse of the SG/MG ratio, the maxima (i.e., SG_dom_ and MG_dom_) in both series occur at different times (Fig 2F inset). The four maxima types differed in their ability to predict avoidance (Fig 2F; F_3,43_ = 10.5, *p* = 2.0 × 10^−4^); only SG_dom_ had predictive power better than chance (t_8_ = 24.56; *p* = 4.0 × 10^−9^). While SG dominance occurred regularly and everywhere and during active and passive behavioral states, it nonetheless predicts successful place avoidance in the immediate future, consistent with SG_dom_ signaling recollection of long-term memories.

### Abnormal recollection in Fmr1-KO mice predicts excessive SG dominance

**Fig 3:**
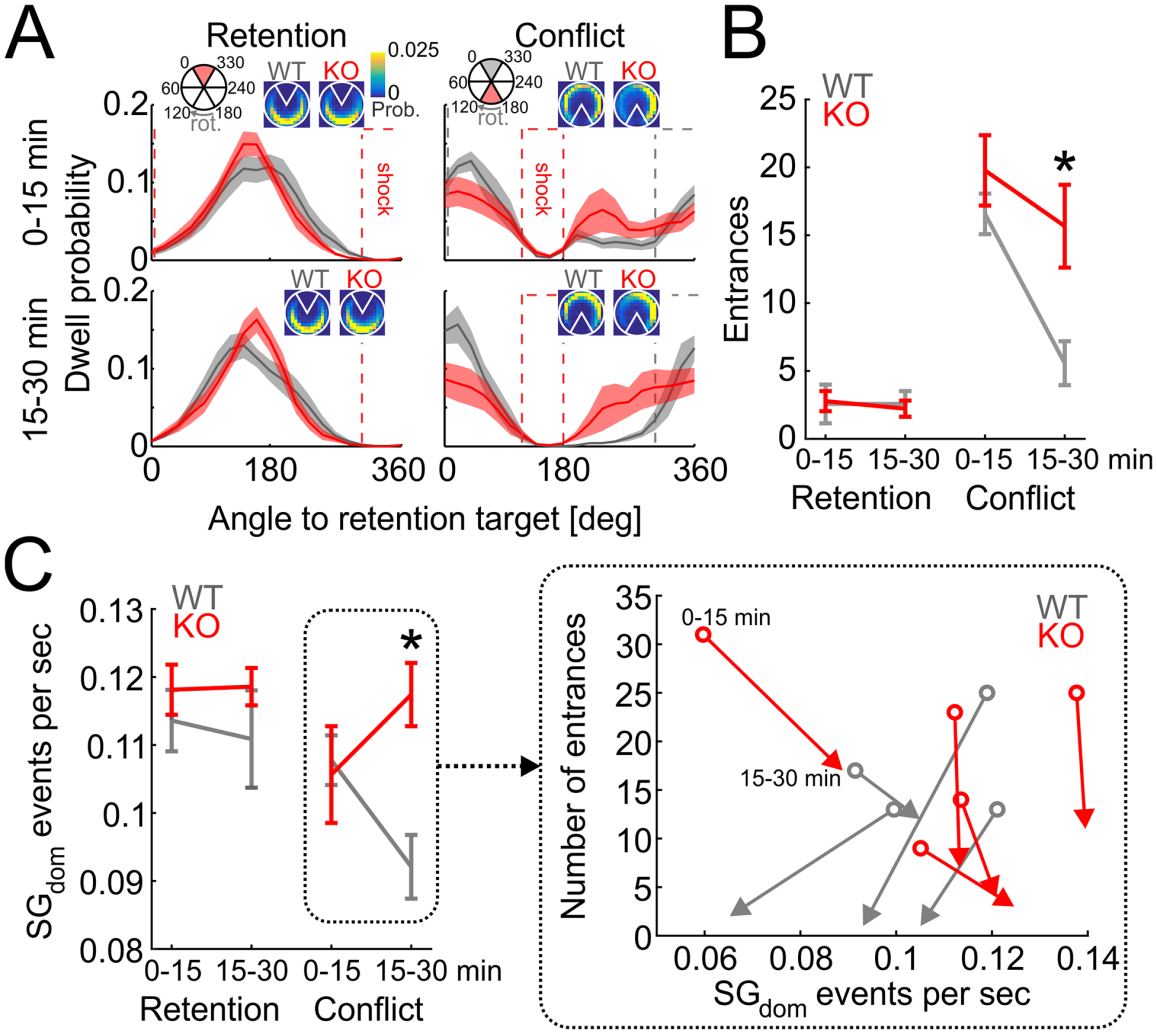
Cognitive inflexibility and associated increases of SG dominance in Fmr1-KO mice. (A) Dwell distribution during first half (0–15 min; top) and second half (15–30 min; bottom) of retention (left) and conflict (right) sessions for WT (gray) and Fmr1-KO (red) mice. Dotted lines show locations of the active shock zone during each session (red) and location of the initial shock zone during conflict sessions (gray). Insets show dwell probability distributions. (B) Behavioral performance during retention and conflict sessions for WT and Fmr1-KO mice. (C) Rates of SG_dom_ events during retention and conflict sessions. *p < 0.05 between genotypes. Data represent average ± SEM. Inset: vectors showing the time evolution from the first half (circles, 0–15 min) to the second half (arrowheads, 15–30 min) of the conflict session in the coordinate system of *x* = SG_dom_ rate and *y* = number of entrances. KO, knockout; rot., rotation; SG, slow gamma; WT, wild-type. *Underlying data can be found here*: [https://goo.gl/oHH22A].

We investigated the hypothesis that SG_dom_ events indicate long-term memory recollection by taking advantage of prior work with Fmr1-knockout (KO) mice [33]. These mice express a null form of the Fmr1 gene to model the genetic defect in FXS, a syndromic form of autism and the most common inherited form of intellectual disability [34]. Place avoidance learning and 24-h retention of long-term memory for the initial shock zone location appears normal in Fmr1-KO mice, but Fmr1-KO mice express cognitive inflexibility when they must avoid the formerly preferred place, because the shock is relocated 180° on a conflict test [33]. We replicated this observation in the mice we recorded (Fig 3A; time spent in six 60°-wide spatial bins during the second half of the conflict session: genotype: F_1,14_ = 4.41, *p* = 0.05; bin: F_5,10_ = 64.26, *p* = 2.7 × 10^−7^; genotype × bin: F_5,10_ = 2.65, *p* = 0.09). Whereas WT mice quickly adapt to the new location of shock on the conflict session, Fmr1-KO mice are impaired, possibly because they persist in recalling the former shock location that is now incorrect (Fig 3B; genotype: F_1,44_ = 6.96, *p* = 0.01; session: F_1,44_ = 77.32, *p* = 3.0 × 10^−11^; time: F_1,44_ = 48.62, *p* = 1.2 × 10^−8^; genotype × session × time: F_1,44_ = 11.16, *p* = 0.002; post hoc tests confirm that WT and Fmr1-KO only differ in the second half of the conflict session).

We then examined if SG dominance distinguishes the WT and Fmr1-KO mice in the conflict session, when the mutants express inflexibility compared to the WT mice. During the initial half of the conflict session, when the genotypes are behaviorally similar, the rate of SG_dom_ events was also indistinguishable between the genotypes. However, the WT SG_dom_ rate decreased in the second half of the conflict session while the Fmr1-KO rate increased, resulting in a significant genotype × time interaction (F_1,24_ = 5.59, *p* = 0.027; Fig 3C) and a marginal genotype × session × time interaction (F_1,24_ = 3.52, *p* = 0.07), because the genotypes only differed on the second half of the conflict session, when WT mice decreased both the number of errors and the rate of SG_dom_ events but the mutant mice did not (Fig 3C right detail). No other effects were significant (F_1,24_’s ≤ 1.71, *p*’s > 0.2). These findings are consistent with the idea that SG dominance reflects recollection of long-term memories and suggest the possibility that Fmr1-KO mice may express abnormally persistent recollection of conditioned place avoidance memory.

### SG dominance predicts nonlocal place coding in neural discharge during active place avoidance

**Fig 4:**
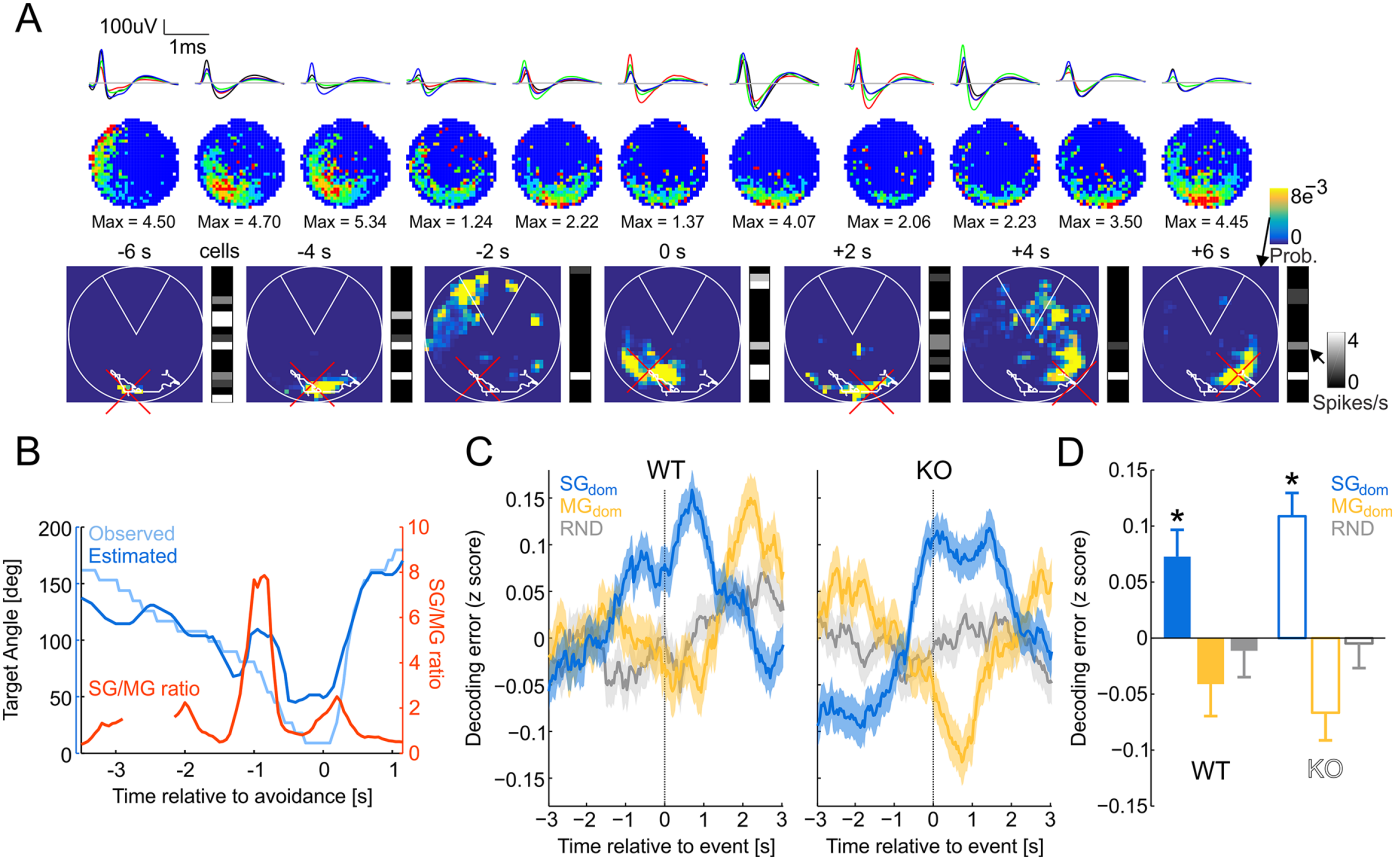
Error in Bayesian decoding of location increases during SG_dom_ events. (A) Example firing-rate maps (top) and 2D decoded Bayesian posterior around avoidance onset. Ensemble activity vectors are shown to the right of each decoded Bayesian posterior. The mouse’s path during a 12-s segment is shown as a white line and the current position is marked by a red cross. (B) Example time series of the angular position that was observed and decoded using a 1D Bayesian estimator from ensemble discharge overlaid with the SG/MG ratio. *T* = 0 s marks avoidance onset. (C) The average of WT and Fmr1-KO z-score normalized 1D decoding error from ensemble activity that is time-locked to SG_dom_ events, MG_dom_ events, and RNDs. *T* = 0 s corresponds to the time of the events. (D) Summary of decoding error at the moments of SG_dom_ events, MG_dom_ events, and RNDs for WT and KO mice. *p < 0.05 relative to random. Data are represented as average ± SEM. KO, knockout; MG, mid-frequency gamma; RND, random time; SG, slow gamma; WT, wild-type. *Underlying data can be found here*: [https://goo.gl/oHH22A].

Next, based on evidence that place cell discharge is more likely to represent nonlocal, distant places during recollection [35–37], we tested the hypothesis that SG dominance identifies recollection. The hypothesis predicts that during the place avoidance task, putative pyramidal cell discharge is nonlocal during SG dominance, assessed as increased error in the location estimate obtained from ensemble firing rates using a Bayesian decoder [38]. We examined CA1 putative pyramidal cell discharge from four WT and three Fmr1-KO mice after initial and conflict avoidance training. For these analyses, the SG_dom_ events were detected independently from all LFP signals recorded at tetrodes on which putative pyramidal cells were identified. We made this decision to avoid bias by choosing one of the tetrodes as representative because detection of SG_dom_ events on all pairs of tetrodes in a given animal was coincident 25% (24.1 ± 18.4% in WT and 25.1 ± 15.8% in Fmr1-KO mice). As predicted, during SG dominance, CA1 putative pyramidal cell discharge decodes to distant locations (Fig 4). The putative pyramidal cell ensemble recorded during active place avoidance (Fig 4A) shows in example Fig 4B that the error between the observed and estimated locations is increased during the SG_dom_ events just prior to avoidance behavior. We computed the average decoding error time-locked to the SG_dom_ events, during which we hypothesize recollection. The decoded error is the z-score normalized average of the 1D posterior probability multiplied by the error function, which was zero at the observed 1D angular location and linearly increased with the distance from the observed location. For comparison, the decoding error was also computed time-locked to random moments as well as relative to MG_dom_ events (Fig 4C). The average decoding error was indistinguishable between the genotypes (WT: 47.5 ± 3.6 degrees; Fmr1-KO: 52.0 ± 4.8 degrees; t_10_ = 0.77, *p* = 0.46). The Bayesian decoding error in both WT and Fmr1-KO mice was large around the time of SG_dom_ events, in contrast to the relatively small error associated with random times (RNDs) and with MG_dom_ events, during which the error was minimal (Fig 4D). Indeed, the decoding errors were greatest during SG_dom_ events (F_2,4215_ = 7.87, *p* = 4 × 10^−4^; Dunnett’s test: SG_dom_ > MG_dom_ = RND), and although this pattern appeared more extreme in Fmr1-KO mice at the time of the event, place representations in Fmr1-KO ensemble discharge did not differ from wild type (genotype: F_1,4215_ = 0.04, *p* = 0.9; genotype × event interaction: F_2,4215_ = 0.33, *p* = 0.72). This result could arise because the Bayesian posterior during SG_dom_ events is less localized and thus more imprecise, or alternatively, during SG_dom_ events, the posterior could be just as compact as during non-SG_dom_ moments, when putative pyramidal cell discharge decodes to current locations.

To control for the possibility that the mouse’s speed might differ during SG_dom_ and random events, we restricted the analysis to only times of stillness (speed < 2 cm/s; 33% of SG_dom_ events). The same pattern was observed, although the effect of genotype became significant because the decoding error was higher in WT during SG_dom_ events, while the decoding error was lower in Fmr1-KO during MG_dom_ events (genotype × oscillation type two-way ANOVA, genotype: F_1,1387_ = 8.38, *p* = 0.004, oscillation type: F_2,1387_ = 22.23, *p* = 3.1 × 10^−10^, interaction: F_2,1387_ = 0.03, *p* = 0.98; Dunnett’s test for difference from random events: SG_dom_: *p* = 0.008, MG_dom_: *p* = 0.049). When analysis was restricted to the times during running (speed ≥ 2 cm/s; 56% of SG_dom_ events), we observed only genetic differences, because Fmr1-KO mice show a higher error of decoding during both SG_dom_ and MG_dom_ events, while WT mice show the same pattern of increased decoding error during SG_dom_ and reduced decoding error during MG_dom_ events (genotype × oscillation type two-way ANOVA, genotype: F_1,2353_ = 7.20, *p* = 0.0073; oscillation type: F_2, 2353_ = 0.81, *p* = 0.45; interaction: F_2, 2353_ = 0.39, *p* = 0.68). The size of the posteriors was indistinguishable during SG_dom_, MG_dom_, and random moments when we decoded 2D position (F_2,2127_ = 0.74, *p* = 0.47). In fact, the posteriors were most compact during SG_dom_, when we decoded the mouse’s 1D angle in the arena relative to the leading edge of the shock zone (F_2,2127_ = 5.04, *p* = 0.006; SG_dom_ < MG_dom_ = RND, according to post hoc Dunnett’s tests), indicating that the nonlocal representations of position during SG_dom_ were compact and precise. These findings confirm that pyramidal cell ensemble discharge selectively represents distant locations during SG dominance, consistent with recollection of locations remote from the mouse.

Because of the role of SWR events in the replay of nonlocal place cell sequences [39], including during fear memory expression [40], we further investigated this nonlocal decoding during isolated SG events (events detected in the 30–50-Hz band without concurrent MG or SWR events). For comparison, we also investigated isolated MG events (events detected in the 70–90-Hz band without concurrent SG or SWR events; see S5 Fig). Approximately 10% of SG and MG events were concurrent with SWR in both genotypes, and so these events were excluded (S5A Fig). Both WT and Fmr1-KO putative pyramidal cell representations appeared more nonlocal during SG events compared to MG events, indicating that events during SWRs cannot account for the observation that SG_dom_ is associated with nonlocal hippocampus place representations (S5C and S5D Fig; genotype: F_1,117515_ = 22.57, *p* = 2.0 × 10^−6^; oscillation: F_2,117515_ = 34.40, *p* = 1.2 × 10^−15^; genotype × oscillation interaction: F_2,117515_ = 3.27, *p* = 0.037; Dunnett’s test for difference from random events: SG_dom_: *p* < 0.0001, MG_dom_: *p* < 0.0001). These statistical tests included the ensemble firing rate as a covariate because of the significant relationships between decoding error and putative pyramidal cell firing rates (WT: *r*^*2*^ = 9%, *p* = 0; Fmr1-KO: *r*^*2*^ = 13%, *p* = 0), whereas the relationships to speed explained substantially less of the variance (WT: *r*^*2*^ = 0.0003%, *p* = 0.69; Fmr1-KO: *r*^*2*^ = 0.4%, *p* = 2.7 × 10^−35^). These findings with isolated SG events as well as those with SG dominance suggest that place memory recollection is predicted by SG dominance, which also identifies when pyramidal cell ensembles will represent remote places, consistent with the hypothesis that SG dominance in hippocampus CA1 identifies active recollection of long-term memory.

### Putative pyramidal cell discharge during SG dominance represents places that will be avoided

**Fig 5:**
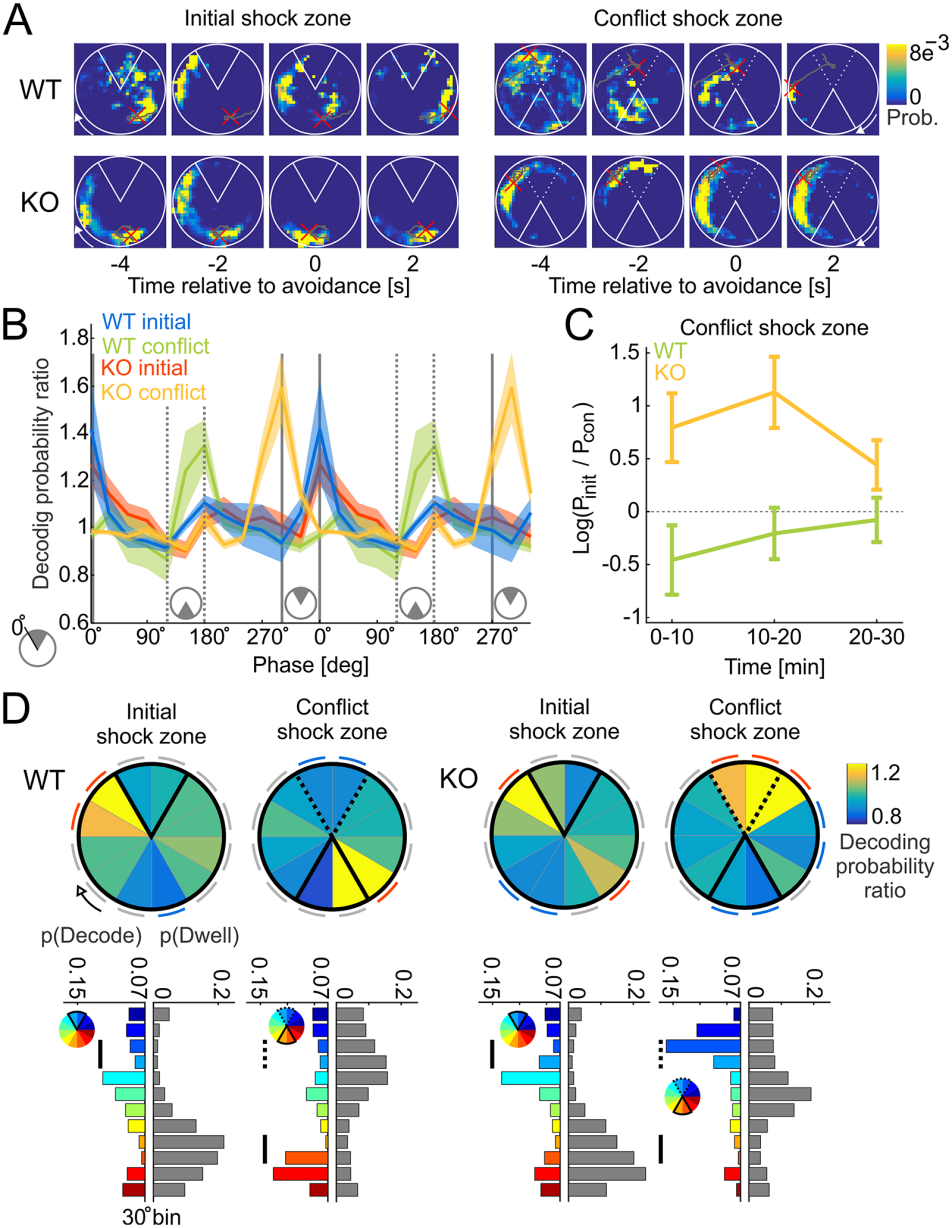
During SG_dom_ events, putative pyramidal cell ensemble discharge represents the vicinity of shock. (A) Four examples of the time evolution of the Bayesian posterior probability from before to after avoiding the shock zone (white sector centered at 12 o’clock for the initial shock zone and 6 o’clock for the conflict shock zone). The mouse’s path during the episode is shown in gray, with the current location indicated by a red cross. The top row corresponds to a WT mouse, the bottom row to a Fmr1-KO mouse. The left examples illustrate training to the initial shock zone. The right examples are after the shock was relocated for conflict training. The initial shock zone location in conflict training is shown as a dotted line. (B) Average normalized posterior probability as a distance from the leading edge of the shock zone. Full gray lines mark the location of the initial shock zone; dotted lines mark the location of the conflict shock zone. Data are represented as averages ± SEM. (C) Ratio of average posterior probability at the location of the initial shock zone and the location of the conflict shock zone in 10-min intervals. (D) Top: summary of normalized posterior probability estimates obtained during SG_dom_ events for the initial and conflict shock zone sessions for WT (left) and Fmr1-KO (right) mice. Notice maximal decoding probability at the leading edge of the shock zone in both WT and KO mice during the initial shock zone session, and during the conflict session, maximal decoding probability is at the leading edge of the relocated shock zone in WT mice but not in Fmr1-KO mice. During conflict, Fmr1-KO ensemble discharge decodes to the vicinity of the initial shock zone that is currently incorrect for avoiding shock. Red arcs located next to angular bins indicate significantly positive (>1) normalized probability (*p* < 0.05), blue arcs indicate significantly negative (<1) normalized probability (*p* < 0.05), gray arcs indicate n.s. relative to 1. Bottom: split bar plots comparing the decoding probability distributions (color: each color corresponds to one 30° angular position) and dwell distributions (gray) for corresponding trials. Black vertical lines mark regions inside of the currently correct shock zone. Dotted vertical lines in conflict trials mark regions inside the initially correct shock zone. KO, knockout; n.s., not significant; P_int_, probability at the initial shock zone location; P_con_, probability at the conflict shock zone location; SG, slow gamma; WT, wild-type. *Underlying data can be found here*: [https://goo.gl/oHH22A].

Finally, we analyzed the Bayesian posterior probability maps from the decoding to examine whether, during avoidance sessions, putative pyramidal cell representations during SG dominance decode to the vicinity of the shock that the mouse will avoid, consistent with recollection of the places to avoid. Fig 5A shows four example Bayesian 2D posterior probability maps computed at times before to times after individual avoidances. There are two examples from each genotype, one when the shock was in the initial location and the other after a conflict session with relocated shock. These examples illustrate that up until about 2 s before the avoidance, the peak values of the posterior probability correspond to the mouse’s location. However, in both genotypes, approximately 2 s before the avoidance of the initial shock location, the posterior probability can peak at nonlocal positions that are in the vicinity of the shock zone or 180° away, which is the safest and most frequented location on the arena during training to the initial shock location. The genotypes differ remarkably in the conflict session. The WT example shows nonlocal decoding to the currently correct, relocated shock location about 2 s before avoidance, whereas the Fmr1-KO example shows decoding to the currently incorrect shock location that was formerly correct.

Similar patterns of representational flexibility in WT and inflexibility in Fmr1-KO are seen in the summary data, computed as the ratio of the posterior during SG_dom_ events normalized by the average posterior during MG_dom_ events, when decoding was local. During SG dominance, this posterior ratio peaks in the vicinity of the initial location of shock, and this is observed for both WT and Fmr1-KO putative pyramidal cell representations (blue and red data in Fig 5B, respectively). The posterior ratio peaks in the vicinity of the currently correct location of shock in the post-conflict session, but only for WT putative pyramidal cell representations, demonstrating representational flexibility (green data in Fig 5B). In the post-conflict session, the Fmr1-KO posterior ratio peaks adjacent to the currently incorrect shock zone (yellow data in Fig 5B). The SG_dom_/MG_dom_ posterior probability ratio averaged across the location of the initial shock zone and the location of the conflict shock zone shows higher decoding probability of the initial shock zone for Fmr1-KO and higher decoding probability towards the conflict shock zone in the WT ensembles (Fig 5C; two-way ANOVA, genotype: F_1,1000_ = 15.44, *p* = 9.1 × 10^−5^; interval: F_2,1000_ = 0.56, *p* = 0.57; genotype × interval: F_2,1000_ = 1.17, *p* = 0.31). The difference in decoding probability became similar in the two genotypes during the 20–30-min interval, consistent with the mice ceasing avoidance behavior when the avoidance is not reinforced. Statistical comparisons of the putative pyramidal cell posterior ratios confirm significant overrepresentation of the regions adjacent to the leading edge of the initial shock zone in WT and Fmr1-KO mice. Overrepresentation is also observed at the relocated shock location in the conflict sessions, but only in WT putative pyramidal cell representations (Fig 5D). In contrast, in Fmr1-KO putative pyramidal cell ensemble representations, the posterior ratios are overrepresented in the currently incorrect shock location during the post-conflict sessions, as confirmed by the significant genotype × trial × region interaction: F_11,36983_ = 4.15, *p* = 3.7 × 10^−6^ (the main effects of genotype and trial were not significant, but the effect of region was significant: F_11,36983_ = 3.68, *p* = 3.0 × 10^−5^; the genotype × region interaction: F_11,36983_ = 2.59, *p* = 0.0027 and trial × region interactions: F_11,36983_ = 4.47, *p* = 2.98 × 10^−5^ were significant as well). Because avoidance behavior of Fmr1-KO and WT mice differed during the conflict sessions (Fig 3A), we also tested the possibility that overrepresentation of the currently incorrect shock zone in Fmr1-KO might be caused by corresponding overrepresentations of where the mice visited. We compared the angular probability distributions of the SG_dom_/MG_dom_ decoding probability ratios and the dwell distributions in the sessions with the initial and conflict shock zones (Fig 5D, bottom) by computing a *t*-distributed test statistic as the absolute difference between the decoding and dwell distributions, divided by the average absolute difference between the first and second halves of each of the decoding and dwell distributions; initial shock zone: WT: t_11_ = 3.54, *p* = 0.002; Fmr1-KO: t_11_ = 2.20, *p* = 0.02; conflict shock zone: WT: t_11_ = 1.80, *p* = 0.04; Fmr1-KO: t_11_ = 2.02, *p* = 0.03). These findings demonstrate that SG dominance corresponds to activation of nonlocal, memory-related pyramidal cell representations and demonstrate, for the first time, representational inflexibility in Fmr1-KO mice concurrent with behavioral inflexibility (Fig 3).

## Discussion

### Summary—A neurophysiological hypothesis for recollection

To identify a neural signature of recollection, we began by selecting an enriched sample of potential recollection events using behavioral criteria (Fig 1) and investigated the rate of occurrence of gamma oscillations in the dorsal CA1 LFP. Individual SG and MG events were not predictive, but SG dominance, i.e., maxima in the slow/mid-frequency ratio of event rates at stratum pyramidale predicted successful place avoidance (Fig 2), suggesting that SG dominance is a candidate neural signature of long-term memory recollection, at least for place memories. While WT mice attenuated SG dominance when it was necessary to suppress recollection of the initially learned locations of shock, SG dominance was not attenuated in Fmr1-KO mice when they demonstrated cognitive inflexibility (Fig 3). SG dominance occurred approximately every 9 s in standard conditions of exploration as well as during place avoidance sessions, which is almost three times more frequent than active avoidance-like behaviors (Fig 2). This indicates that if SG dominance corresponds to an internal variable like recollection, then it may not merely be the recollection of conditioning events such as locations of shock. Indeed, SG dominance coincides with nonlocal putative pyramidal cell representations during post-avoidance recording sessions (Fig 4). We note that, although Fmr1-KO mice model the genetic defect in FXS and express a number of biochemical and synaptic abnormalities [41,42], their putative pyramidal cells that are classified as place cells express normal place fields [43], which makes their cognitive flexibility deficits a challenge to explain. However, guided by the notion that SG dominance identifies long-term place memory recollection, we observed neural representational inflexibility in Fmr1-KO mice when they express behavioral inflexibility (Fig 5). Together, these findings provide convergent evidence that SG dominance predicts recollection as well as abnormalities of recollection in Fmr1-KO mice.

### Summary—Considering alternative interpretations

Recollection is an internal variable, inaccessible to direct observation; thus, alternative accounts for SG dominance were considered, but none are compatible with all the observations. The findings are incompatible with the alternative possibility that SG dominance merely indicates a process that anticipates or prepares to initiate movement (Fig 2). Movement preparation might have appeared to account for SG dominance, because we initially identified SG_dom_ events by assessing avoidance behavior that was characterized by a period of stillness before the mouse moved away from the location of shock. However, after recognizing that SG dominance might be a neural correlate of recollection, we investigated alternative accounts like avoidance behavior itself, movement preparation, and other speed-related possibilities. While successful avoidances are most frequent about 1.5 s after SG_dom_ events (Fig 2E), SG_dom_ events are far more frequent and distributed distinctively from active avoidance movements (Fig 2B). Although SG_dom_ events are similarly present during continuous immobility and running, their likelihood is overrepresented during stillness and transitions from stillness to running, compared to randomly selected moments (Fig 2D). During pretraining, before the mice experienced shock, the prevalence of movement-related behaviors during SG_dom_ events did not differ from the prevalence of these behaviors during random events (S4B Fig). Thus, mere movement-related classifications of behavior do not account for when SG dominance is expressed. The deviations from chance expectations after place memory training suggest that an internal cognitive variable may be influencing the expression of SG dominance. Nonetheless, because movement conditions vary with SG dominance, we considered other known movement-related effects on gamma oscillations. Gamma frequency increases with running speed [44], more for faster than SG frequencies [45]; however, SG_dom_ is distinctive, characterized by relatively increased rather than decreased SG power (Fig 1D). During steady immobility prior to avoidance movements, slow and fast gamma oscillations change, and they change differently, indicating that any relationship to speed is complex and not explained by known relationships to speed (S3E Fig). Similarly, because SG_dom_ events are associated with nonlocal place representations (Fig 4, S5 Fig), they are unlike the events during the recently described N-waves that are associated with local place representations during immobility [46]. Furthermore, after isolating SG oscillatory events from contamination by MG events and SWRs and correcting for firing-rate bias of the decoding, SG events still expressed nonlocal decoding. Correspondingly, local place representations are predominantly observed during MG_dom_ events, when SG is relatively weak (Fig 4, S5 Fig). Consequently, SG_dom_ events may represent a complementary network state because, in particular, unlike N-waves, the discharge associated with SG_dom_ events is nonlocal (Fig 4, S5 Fig). This is consistent with SG dominance signaling recollection of long-term memories of remote places and/or spatial events. By inspection, despite the nonlocal hippocampal representational discharge, vicarious trial and error (VTE) [47–49] did not coincide with SG dominance. This was confirmed by statistical comparisons of numerical estimates of VTE [49] during SG dominance, MG dominance, and random events (S6G Fig; ANOVA F_2,9857_ = 0.1, *p* = 0.9). Putative pyramidal cell discharge is also nonlocal in SWR events, during which sequences of place cell discharge from active behavior can be observed to replay [50–53]. This sharp-wave associated replay is thought to underlie memory consolidation and support memory and decision-making during the initial stages of learning [46,54,55]. Because SG_dom_ events are distinct from this sharp-wave associated discharge (S5 Fig), the SG_dom_ events represent different phenomena. Thus, the present findings of SG dominance and its associations to cognitive variables are distinct from known relationships of gamma to hippocampal network phenomena and to overt behavioral variables. Accordingly, based on the present findings, we contend that SG dominance in the CA1 stratum pyramidale LFP is a neural signal that the hippocampus network is in the functional mode of recollecting long-term memories that are used to guide behavior, as in standard tests of long-term memory.

### Implications for the routing-by-synchrony hypothesis

Identifying SG dominance as a neural signal of recollection was inspired by prior work that proposed SG oscillations measured at stratum pyramidale correspond to recollection event–associated activity from the CA3 region into stratum radiatum, whereas the neocortical inputs to the stratum lacunosum-moleculare are associated with higher frequency gamma and carry information about what is currently being experienced for encoding [7,12,13]. This important idea offers a solution for how multiple types of place information might be routed to the hippocampus to be used judiciously [8,36,56], for example, to solve place avoidance tasks on a rotating arena, during which distinct representations of the same physical places are activated [57,58]. However, careful analysis of the extracellular currents along the dendritic compartments of dorsal CA1 has not supported this basic proposition [59,60]. CA1 gamma-organized spiking is not simply entrained to the gamma organization of the inputs from CA3 and entorhinal cortex, and the discrete oscillatory events at the stratum radiatum and stratum lacunosum-moleculare compartments have frequency components that overlap in the SG and MG frequency bands (S2 Fig) [14,16,27,33,61]. Furthermore, these inputs are also relatively tonic, which has been both estimated [62] and observed during behavior [63,64]. One factor that might add difficulty when interpreting differences in the literature is that most studies assume steady-state cognitive conditions, which is not the case during either place avoidance or the foraging tasks that are often used, despite physical steady-state conditions [57,58,65–67]. By selecting cognitively homogeneous samples, we find that recollection of hippocampus-dependent, actionable information is marked by the perisomatic region of CA1 being dominated by SG over MG oscillations, as if the two signals are in continuous competition (see S2 Fig). These SG dominance “recollection” events appear to require a relatively large decrease in MG, in coordination with a lesser or no decrease in SG, possibly depending on task conditions (Fig 1). Because MG-associated entorhinal inputs facilitate CA1 spiking [14,16], these observations point to a role for regulation of feed-forward inhibition in the competition between temporoammonic and Schaffer collateral inputs to CA1 [16,27,68–70]. The present observations suggest that recollection of long-term memory is a transient change in the balance of the two signals that is rapidly followed by a reversal of the dominance of MG by SG. This result contrasts previous studies suggesting the appearance of one or the other type of gamma during processes of encoding and retrieval [7,12,13]. Contradictory observations that SG and MG are commonly observed in the same theta cycle (S3 Fig) [14,27] can be explained by the analyses that show that the previously used thresholds of oscillation power selects for separate slow- and mid-frequency–preferring theta cycles (S3 Fig). While the present data do not support the details of the routing-by-synchrony hypothesis as first proposed [12,13], the present findings support the gist, but without common feed-forward conceptions. Rather, this work has revealed dynamical operations within near-continuous arrival of oscillation-associated inputs along the somato-dendritic compartments of CA1 [63,64,69]. This input engages excitation, inhibition, and disinhibition and is integrated locally in dendrites, such that the discharge of CA1 principal cells occurs as if embedded within a local neural activity infrastructure, from which their spiking can emerge when the local infrastructure permits, by its transient adjustments to create distinctive information-processing modes, like encoding and recollection [61,63,71–75]. These transient adjustments appear to emerge through a complex interplay between local neural dynamics and afferent activity [56,60,69,76], and while the rules of engagement for this competition remain unknown, they are neither merely nor predominantly feed forward, in part because the gamma oscillations are locally generated and not dependent on gamma-paced inputs, especially in the MG and high gamma frequency ranges [16,27].

### A neural signature of recollection

The recollection events we identified as perisomatic SG_dom_ events are brief, and they recur after several seconds, which may be a candidate mechanism for the seconds-long overdispersion dynamics in place representations that have been observed in single-unit place cell ensemble studies during both place-responding and foraging behaviors with no explicit cognitive demand [57,58,65,66,77]. Because the timescales differ and the SG_dom_ events span several theta cycles, they are unlikely to be the same cognitive information-processing mechanism that governs the sub-second dynamics of single place cell spiking that can be observed as rodents traverse a cell’s place field, which is interpreted as a form of encoding and recollection [7,60,78]. Rather, the SG_dom_ events suggest that cognitive information processing is intimately tied to the coordinated regulation of inhibition at the perisomatic region and perhaps elsewhere, under the control of the distinct, anatomically segregated, information-carrying afferents to CA1 [56,61,79], although the anatomical segregation of inputs may not be a requirement [69]. Resembling neural control of incompatible behaviors in leech [80], the present findings demonstrate in gamma, a specific, dynamic coordination of excitation and inhibition that controls the cognitive information processing that permits effective spatial cognition, whereas its discoordination is associated with cognitive inflexibility [33,56,81]. Alterations in this coordination account for the cognitive effort of animal subjects both when they demonstrate adaptive cognitive information processing [14,82] and when they exhibit inflexible cognition, as was observed in both the neural signals and the behavior of the WT and the Fmr1-KO mouse model of FXS and autism-related intellectual disability [33].

## Materials and methods

### Ethics statement

All methods comply with the Public Health Service Policy on Humane Care and Use of Laboratory Animals and were approved by the New York University Animal Welfare Committee (animal protocols 12–1386 and 13–1427) and the State University of New York, Downstate Medical Center Institutional Animal Care and Use Committee (animal protocol 11–10265). Because detailed methods have been described [31,33], only brief descriptions are provided.

### Subjects

A total of 21 WT mice with a mixed C57BL6/FVB background were used as well as 20 Fmr1-KO mice carrying the Fmr1^tm1Cgr^ allele on the same mixed C57BL6/FVB background. The mutant mice were obtained from Jackson Laboratories (Bar Harbor, ME) to establish local colonies. The mice were 3–6 months old. LFP recordings and behavior from 16 WT and 17 Fmr1-KO mice animals were studied in [33]. Of those mice, nine WT mice were recorded during avoidance training, with electrodes localized in stratum pyramidale and used for analyses in Figs 1 and 2. Seven WT mice and nine Fmr1-KO mice were used for behavioral analyses of 24-h retention of place avoidance and conflict training (Fig 3). Four WT mice and five Fmr1-KO mice with electrodes localized in stratum pyramidale were recorded during conflict training and used in electrophysiological analysis in Fig 3. Four WT mice and three Fmr1-KO mice were implanted with tetrodes and recorded after place avoidance training. These were used for the analyses in Figs 4 and 5. One mouse was used for the CSD analysis in S2 Fig.

### Surgery to implant electrodes

The LFP recordings from the 16 WT and 17 KO mice that were previously analyzed [33] were made from a bundle of six 75-μm Formvar-insulated NiCh wire electrodes (California Fine Wire, Grover Beach, CA), staggered by 170 μm, that were stereotaxically implanted under Nembutal anesthesia (60 mg/kg i.p.). The tip was aimed at −1.80 AP, ±1.30 ML, −1.65 DV, relative to bregma. The electrodes spanned the dorsoventral axis of the dorsal hippocampus, but the spacing was too great for current-source-density analyses. Reference electrodes were aimed at the cerebellar white matter. For single-unit recordings from four WT and three Fmr1-KO mice, an 8-tetrode flexDrive (www.open-ephys.org) or a 4-tetrode custom drive was implanted under isoflurane anesthesia (2%, 1 L/min), with the electrodes aimed at the dorsal hippocampus [83], and bone screws, one of which served as a ground electrode. The electrode assemblies were anchored to the skull and bone screws with one of two dental cements (Grip Cement, Dentsply, Milford, DE, and TEETs Denture Material, Co-oral-ite Dental MMG, Diamond Springs, CA). The mice were allowed at least one week to recover before experiments began.

### Electrophysiological recording

A custom unity-gain buffering preamplifier was connected to the electrode assembly, and the electrophysiological signals were galvanically transmitted to the recording system by a lightweight counterbalanced cable. The differential signal from each electrode was low-pass filtered (600 Hz) and digitized at 2 kHz for LFPs and band-pass filtered (500 Hz–6 kHz) and digitized at 48 kHz for action potentials using dacqUSB (Axona, St. Albans, UK). Two-millisecond duration tetrode action potential waveforms were collected and stored for offline single-unit isolation using custom software (Wclust; see S6 Fig). Single-unit isolation quality was quantified by computing IsoI_BG_ and IsoI_NN_ [84]. Single units (*N* = 455) were recorded and analyzed from 6 WT and 3 Fmr1-KO mice. Only 213 single units with both IsoI_BG_ and IsoI_NN_ greater than 4 bits were judged to be well isolated, and 163 of these were of sufficiently high-quality putative pyramidal cells that express place cell or nonspatial discharge characteristics, according to objective criteria (see S6 Fig). LFPs were localized as previously described [33] or to CA1 stratum pyramidale because they showed LFP activity characteristic of stratum pyramidale and were recorded by the same electrode as place cells. The electrode locations were verified histologically at the end of recordings.

### Active place avoidance

The active place avoidance task has been described in detail [85,86] and the behavioral protocol was identical to [33]. Briefly, the mouse’s position was tracked 30 times a second using an overhead camera and a PC-based video tracking system (Tracker, Bio-Signal Group, MA). All sessions lasted 30 minutes. Pretraining on the rotating (1 rpm) arena was followed 24 h afterwards by three training sessions, during which the mouse (*n* = 40) received a mild 0.2-mA, 60-Hz, 500-ms foot shock whenever it entered the shock zone. There was a 2-h rest in the home cage between training sessions. A subset of the mice received conflict training (*n* = 14), in which the conditions were identical to the training phase except that the shock zone was on the opposite side. The conflict task variant tests cognitive flexibility, because the mice should suppress recollection of the initial memories of the location of shock so they can learn and use the new location of shock. Note that because the shock zone is unmarked, the physical conditions of all the sessions are identical except when the mouse is shocked, which is rare; for example, only for 10 s if a mouse receives 20 shocks during a 30-min session.

### Data analysis

#### Detection of behavioral events

During the first session of the initial training on the rotating arena, spatial behavior becomes stereotyped, with periods of stillness when the mouse is passively carried towards the shock zone by the arena’s rotation and periods of movement directed away from the shock zone. This is quantified when angular distance to the shock zone is plotted against time; it reveals a sawtooth profile (Fig 1B, top). We selected two behavioral events based on the angular distance to the shock zone. The onset of avoidance (blue dots in Fig 1B, top) was defined as local minima in the target angle time series with preceding stillness, without entering the shock zone. The second event was an escape (red dots in Fig 1B, top), defined as entrance to the shock zone with preceding stillness. To define stillness, speed was computed using a 400-ms-long sliding window. Stillness was identified as intervals with speed below 2 cm/s. Brief crossings of the stillness threshold for less than 150 ms were not considered departures from stillness. Because some avoidances were preceded with the animal’s initial acceleration towards the shock zone followed by a turn, local minima in the target angle time series occurred during speed above the stillness threshold; we included all avoidances with at least 1 s of stillness in a 3-s window prior to the detected avoidance onset.

#### Quantifying VTE

VTE was quantified during SG_dom_, MG_dom_, and randomly selected events, as in prior work [49]. Briefly, the orientation of motion (phi) was first calculated from the position time series as the arctangent of the change in x- and y-coordinates across 33-ms time steps. The change in phi across 33 ms was then calculated to represent the angular acceleration of motion time series. The absolute value of this time series was summed in a 500-ms window during an event to estimate the amount of head sweeping in the event.

#### Preprocessing for LFP recording quality

The LFP data were first processed by a custom artifact rejection algorithm, which marked continuous segments of artifact-free signals. Such segments that were 4 s or longer were used for further analysis. The majority of artifacts were related to the foot shock—specifically, signal saturations and slowly changing DC signals, as the recording system recovered from the shock artifact. Constant signals close to the maximal voltage of the analog-digital convertor defined signal saturation. Periods of very low signal difference defined the slowly changing DC signal artifacts. Thresholds for an acceptable signal difference were selected by visual inspection and used for analysis of the entire data set. Each artifact segment was extended by 0.25 s on both sides, and all artifact-free segments smaller than a 1-s minimum window were also discarded. Each channel in the data set was processed independently, and the algorithm’s performance was verified by visual inspection.

#### Detection of oscillatory events

A published algorithm was used to extract oscillatory events from the LFP independently for each recorded channel [31]. In the first step of the algorithm, the LFP is transformed into a time-frequency power representation by convolving the LFP signal *S* with a group of complex Morlet wavelets, resulting in complex band-specific representations *S*_*f1*_–S_*fN*_, where *f*_*1*_–*f*_*N*_ represent frequencies of interest (Fig 1E). Instantaneous normalized power is then obtained by squaring the argument of the band-specific signal representation and dividing it by the square of the standard deviation of the band-specific signal, such as $\frac{{\left|X_{fi}\right|}^{2}}{{std(X_{fi})}^{2}}$. In the next step, oscillatory events are detected as local peaks in the normalized 2D time-frequency space (S2E Fig). Band-specific oscillation rates are then computed as the number of detected events in representative frequency bands (30–50 Hz for SG, 70–90 Hz for MG), with power exceeding a defined threshold (2.5 SD of the mean power) per unit of time (refer to S3 Fig for the rationale for selecting power thresholds and representative frequency bands).

#### Calculation of instantaneous SG/MG ratio

First, we extracted band-specific oscillatory rates in 1-s-long windows advanced by 0.25 s. Next, we smoothed the estimated rates of oscillatory events by 2.5-s-long windows and took the ratio of SG (30–50 Hz) to MG (70–90 Hz) oscillatory rates. To obtain the maxima of the SG/MG ratio (SG_dom_ events), we searched for local maxima in the SG/MG series with peak prominence (amplitude difference between the maxima and preceding and following minima) of at least 1 and amplitude above 1 (corresponding to SG > MG). SG/MG minima (MG_dom_ events) were obtained in the same way by finding local maxima in the inverse MG/SG time series using the same prominence and amplitude settings (corresponding to SG < MG).

#### Bayesian analysis

To obtain estimates of the mouse’s location based on single-unit data, we used a published algorithm [38], in which the probability of the current location is defined as $P(x|n)=C\left(\tau ,n\right)P\left(x\right)\left(\prod_{i=1}^{N}f_{i}{\left(x\right)}^{n_{i}}\right)\text{exp}\left(-\tau \sum_{i=1}^{N}f_{i}\left(x\right)\right)$, where *C*(*τ*, ***n***) is a normalization factor, so that ∑_*x*_*P*(***x***|***n***) = 1, *f*_*i*_(***x***) are firing-rate maps for cells *i–N* obtained either by binning the 2D space into 32 × 32 bins or 1D space (distance to shock zone) into 20 or 12 angular bins; *P*(***x***) is the dwell distribution; *τ* is the length of the time window (500 or 200 ms); *n*_*i*_ is the number of spikes fired by the *i*-th cell in a given time window; and *x* is the *(x*,*y)* position of the animal in the 2D analysis or the angular position in the 1D analysis. Only recordings with at least five high-quality spatial or nonspatial putative pyramidal cells were analyzed. Time windows with no spikes were excluded from analysis. To obtain the error of the location estimate in 1D, we created a linear error function *E*, which was zero at the observed location, and at any other location, it corresponded to the distance from the observed location such that *E* = *d*(*x*_*obs*_, *x*_*decode*_), where *d* is the distance between the decoded angular bin *X*_*decode*_ and bin, *X*_*obs*_, where the mouse was observed. We then multiplied the location estimate *P*(***x***|***n***) by the error function and took the average, so that location errors at the highest probability contributed proportionately more to the resulting error estimate. Considering the entire location estimate *P*(***x***|***n***) instead of only its maximum leads to a lower overall decoding error and was therefore used throughout this study. The resulting error estimate was z-score normalized to account for absolute differences in the decoded error due to different numbers of putative pyramidal cells in a given recording.

#### Statistical analysis

Statistical analyses were performed using JMP version 12 (SAS, Cary, NC) and Matlab 2016b (Mathworks, Natick, MA). Significance was accepted at *p* < 0.05. Exact *p*-values are reported throughout; *p* is reported as 0 when *p* < 10^−125^.

## Supporting information

S1 Video Related to Fig 2; dynamical schematic representation of SG_dom_-identified recollection. Real-time slow (30–50 Hz; low pitch) and mid-frequency (70–90 Hz; high pitch) gamma event replay represented as an audio track. Notice how both SG and MG events co-occur but that SG events can dominate. Top, left: polar coordinate of the mouse in a 55-s-long window (0° corresponding to the leading edge of the shock zone). The current time in the video is indicated by the vertical line. Middle, left: SG and MG rates. Bottom, left: SG/MG ratio. Top, right: location of the mouse in the place avoidance environment with the shock zone shown in red. The current location is indicated in black and the prior 6 s (180 samples) of locations are shown in gray scale from black to white. Bottom, right: time-frequency representation of the LFP obtained from stratum pyramidale in the 25–100-Hz range. LFP, local field potential; MG, mid-frequency gamma; SG, slow gamma.
(MP4)

S1 Fig Related to Fig 1; basic LFP properties during active avoidance. (A) Power spectra with log frequency axis during periods of stillness and running throughout active avoidance training. Dotted lines indicate linear fits to the data. (B) Power spectra with linear frequency axis during periods of stillness and running throughout active avoidance training. (C) SWR rates during pre-avoidance stillness and overall stillness. LFP, local field potential; SWR, sharp-wave ripple. *Underlying data can be found here*: [https://goo.gl/oHH22A].
(TIF)

S2 Fig Related to Fig 1; oscillatory activity in mouse dorsal hippocampus CA1 LFPs. (A) LFP signals obtained using 32-site linear silicon electrode array during an SWR. (B) CSD analysis of CA1 LFP to separate individual oscillatory components within CA1. (C) CSD power profiles averaged over theta cycles from s.p., s.r., and s.l.m electrodes. Notice the mixture of three gamma types (SG, 30–50 Hz; MG, 60–100 Hz; and fast gamma, >100 Hz) at the s.p. electrode. (D) Normalized LFP power from the s.p. electrode averaged across theta cycles for running (speed ≥ 2 cm/s) and stillness (speed < 2 cm/s). (E) Example: 1.5-s LFP obtained from the s.p. electrode (white) and its time-frequency representation obtained by wavelet transform. Each frequency band was normalized separately by dividing signal power with signal variance. Notice the presence of mixed states when SG and MG oscillations are present in a single theta cycle. Individual theta cycles are marked by vertical lines. Oscillatory events detected as local maxima in time-frequency 2D space with peak power >2.5 SD are marked with red crosses. CA1, Cornu Ammonis; CSD, current source density; LFP, local field potential; MG, mid-frequency gamma; SG, slow gamma; s.l.m., stratum lacunosum moleculare; s.p., stratum pyramidale; s.r., stratum radiatum; SWR, sharp-wave ripple. *Underlying data can be found here*: [https://goo.gl/oHH22A].
(TIF)

S3 Fig Related to Fig 1; selecting the threshold for oscillatory events and selecting the oscillation frequencies to represent slow and mid-frequency gamma. (A) Example time-frequency representation of LFP before and after avoidance. Power in the mid-frequency gamma range during stillness prior to avoidance is typically attenuated, leading to a higher number of detected low power events (*N* = 13, red rectangle around *T* = −1 s). Power in both slow and mid-frequency gamma ranges is typically increased during running away from the shock zone, leading to a higher number of detected high power events (*N* = 7, red rectangle around *T* = +0.75 s). (B) Average normalized power in a 1-s interval is negatively correlated with the number of low power events (z < 1) in the interval. (C) The proportion of detected events after applying different power thresholds. (D) Top: ratio of detected theta cycles with only S, M, S/M, and no detected oscillations (Ø) for power threshold z > 0 (left) and z > 4 (right). Bottom: relationship between the power threshold and the ratio of theta cycles with a single type of oscillation (slow or mid-frequency gamma) and the ratio of theta cycles with mixed oscillations (slow and mid-frequency gamma; black), plotted together with the number of supra-threshold oscillations per theta cycle (blue). (E) The average oscillation rates for 20-Hz wide bands covering the 20–110-Hz frequency range around avoidance onset (*T* = 0) for power thresholds z ≥ 1, 2, 2.5, and 3. Only the average profiles are included for clarity. LFP, local field potential; M, mid-frequency gamma; S, slow gamma; S/M, mixture of slow and mid-frequency gamma. *Underlying data can be found here*: [https://goo.gl/oHH22A].
(TIF)

S4 FigRelated to Fig 3; avoidance histograms during retention and conflict sessions, probability of behavioral states during SG_dom_ and random events in pretraining sessions and SG_dom_ event rates across training sessions.(A) Locations of avoidances across six 60° sectors during the first and second halves of the memory retention and conflict sessions. Avoidance profiles analyzed by two-way ANOVA with repeated measures; only the second half of the conflict session (15–30 minutes; S4A Fig bottom, right) shows a significant genotype × bin interaction (F_5,8_ = 4.56, *p* = 0.03), as Fmr1-KO animals display stronger avoidance of locations associated with the initial shock zone (0–60°, 300–360°). (B) Proportions of different behavioral events detected during SG_dom_ events during pretraining sessions before ever experiencing shock (filled bars) compared to randomly selected events (empty bars; comparisons of SG_dom_ to Random Still: $\chi_{1}^{2}=6.5$, *p* = 0.16; Run: $\chi_{1}^{2}=8.3,$*p* = 0.08; Still→Run: $\chi_{1}^{2}=9.3$, *p* = 0.05; Run→Still: $\chi_{1}^{2}=0.004$, *p* = 0.99). (C) Average SG_dom_ rates across initial 15 min of first and last training sessions (two-way ANOVA with repeated measures genotype × trial: genotype: F_1,13_ = 0.30, *p* = 0.59; trial: F_1,13_ = 6.91, *p* = 0.02; genotype × trial: F_1,13_ = 0.04, *p* = 0.85). KO, knockout; SG, slow gamma. *Underlying data can be found here*: [https://goo.gl/oHH22A].(TIF)

S5 Fig Related to Fig 4; nonlocal place representations in putative pyramidal cell ensemble discharge during SG oscillations without concurrent MG oscillations and SWRs in WT and Fmr1-KO mice. (A) Average voltage traces during isolated SG, MG, and SWR events. Percentage of (non-isolated) SG and MG events that coincide with SWR events. (B) Relationships between location decoding error (smaller error = more accurate) and running speed (top) and ensemble firing rate (bottom). (C) Bayesian decoding error during SG oscillations that are not accompanied by MG oscillations or SWRs (blue), MG oscillations that are not accompanied by SG oscillations or SWRs (yellow), and random events that are not accompanied by SWRs (gray) in WT and KO mice, corrected for firing-rate bias of the decoding. (D) Summary of decoding error during isolated SG and MG oscillations and random events. *p < 0.05 relative to random events. KO, knockout; MG, mid-frequency gamma; SG, slow gamma; SWR, sharp-wave ripple; WT, wild-type. *Underlying data can be found here*: [https://goo.gl/oHH22A].
(TIF)

S6 Fig Related to Fig 4; single-unit recordings and selection of the pyramidal cell data set. (A) Example of single-unit isolation and the Open Ephys microdrive (left inset) and an implanted mouse (right inset). The inset table lists isolated units with their corresponding *IsoI*_*BG*_ and *IsoI*_*NN*_ values used for selecting units with sufficient isolation quality. Colors in the table correspond to clusters on the left. Only units with both quality measures >4.0 were analyzed further. (B) Neuronal subtype classification into three subtypes representing putative pyramidal cells with spatial specificity (type 1; blue), putative pyramidal cells without spatial specificity (type 2; green), and putative interneurons (red). Each dot represents a single well-isolated unit. Plot in 2D principal component space computed from the original 7D feature space that describes each unit. These features are the largest spike’s width, the unit’s firing rate, proportion of active pixels, firing-rate map coherence, firing-rate map information content, peak ISI, and proportion of spikes in a burst (≤10 ms ISI). (C) Histology showing electrode placement in CA1. Red and black ellipses mark tip of tetrode and point of entering cortex, respectively. (D) Example spatial putative pyramidal cells, nonspatial putative pyramidal cells, and putative interneurons from example wild-type (top row) and Fmr1-KO (bottom row) animals. (E) A seven-cell ensemble of spatially tuned putative pyramidal cells with their corresponding firing-rate maps (left) and raster plots of firing (top, right) during 100 s. The corresponding SG/MG ratio is shown in red (bottom, right), with LFP waveforms around the SG/MG maxima and minima, with identified SG (blue arrows) and MG (yellow arrows) oscillatory events. (F) Theta (8-Hz) phase preference of spatially tuned putative pyramidal cell discharge for wild-type (gray) and Fmr1-KO (red) mice. (G) Vicarious trial-and-error score computed for SG_dom_, MG_dom_, and random events. CA1, Cornu Ammonis 1; ISI, inter-spike interval; KO, knockout; MG, mid-frequency gamma; SG, slow gamma. *Underlying data can be found here*: [https://goo.gl/oHH22A].
(TIF)

S7 Fig Related to S1 Video; raw LFP and wavelet spectrum examples of identified SG_dom_ episodes. (A) SG/MG ratio computed over a 55-s-long time period, with identified SG_dom_ peaks (gray circles) and a selected subset of SG_dom_ peaks used for LFP and wavelet spectrum extraction in the examples below (red circles). (B–D) LFP (top) and wavelet spectrum (bottom) of 1-s-long segments centered on SG_dom_ peaks. Power peaks with z-score power >2.5 were marked by a white cross. LFP, local field potential, MG, mid-frequency gamma; SG, slow gamma. *Underlying data can be found here*: [https://goo.gl/oHH22A].
(TIF)

S1 Text (DOCX)

S2 Text (DOCX)

S3 Text (DOCX)

## Abbreviations

CA1: Cornu Ammonis 1
CA3: Cornu Ammonis 3
CSD: current source density
ECIII: entorhinal cortex layer 3
EEG: electroencephalogram
FXS: Fragile X Syndrome
ING: interneuron network gamma
ISI: inter-spike interval
KO: knockout
LFP: local field potential
MG: mid-frequency gamma
n.s.: not significant
phi: orientation of motion
PING: pyramidal interneuron network gamma
RND: random time
s.l.m.: stratum lacunosum moleculare
s.p.: stratum pyramidale
s.r.: stratum radiatum
SG: slow gamma
SG_dom_: slow gamma dominance
SWR: sharp-wave ripple
VTE: vicarious trial and error
WT: wild-type
