# Supplementary figures and images for: Control of recollection by slow gamma dominating mid-frequency gamma in hippocampus CA1
Source: PLoS Biol. 2018 Jan 18;16(1):e2003354. doi: 10.1371/journal.pbio.2003354 (PMC5790293; doi:10.1371/journal.pbio.2003354)

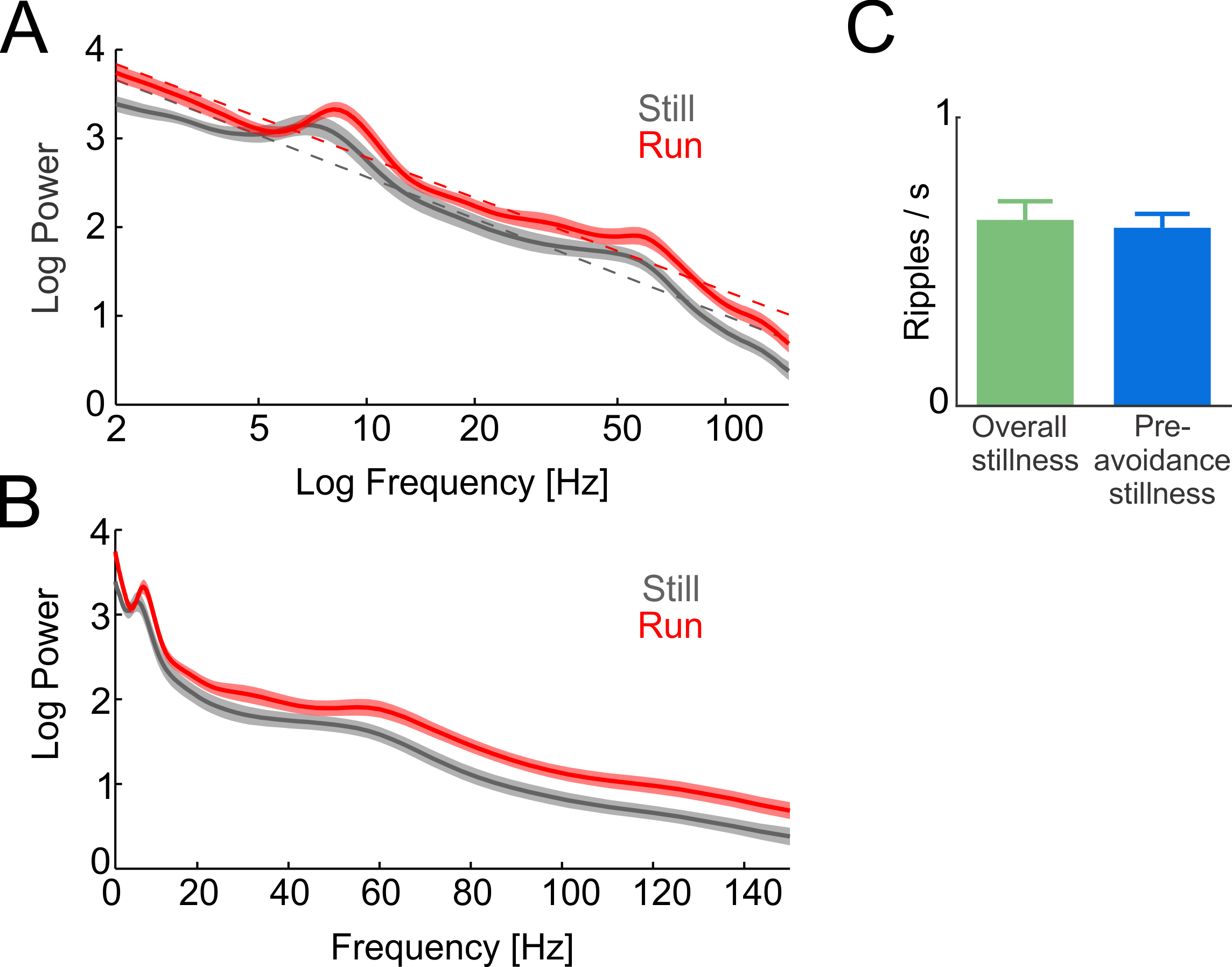

Supplement: S1 Fig — (A) Power spectra with log frequency axis during periods of stillness and running throughout active avoidance training. Dotted lines indicate linear fits to the data. (B) Power spectra with linear frequency axis during periods of stillness and running throughout active avoidance training. (C) SWR rates during pre-avoidance stillness and overall stillness. LFP, local field potential; SWR, sharp-wave ripple. Underlying data can be found here: [https://goo.gl/oHH22A]. (TIF) [file pbio.2003354.s002.tif]

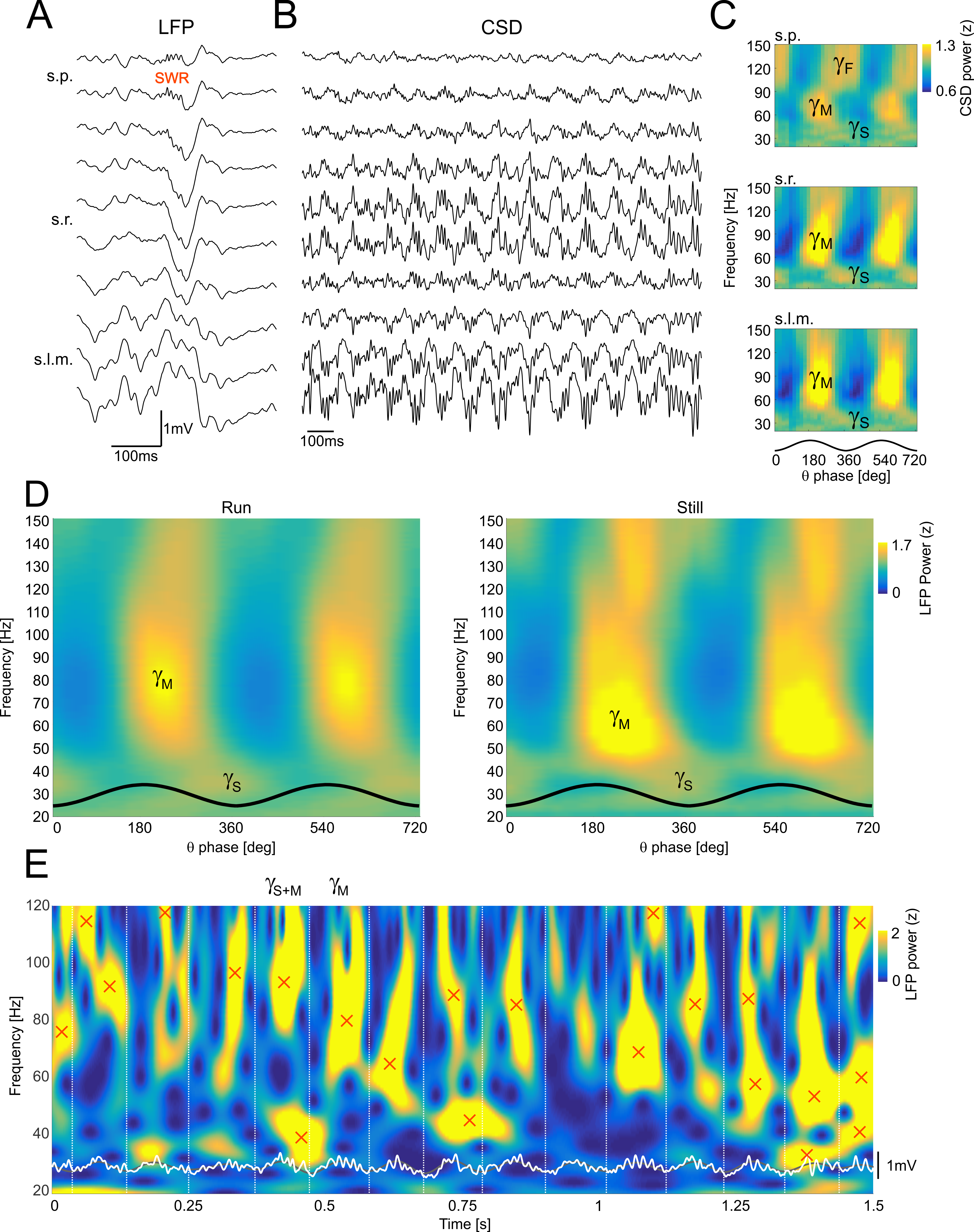

Supplement: S2 Fig — (A) LFP signals obtained using 32-site linear silicon electrode array during an SWR. (B) CSD analysis of CA1 LFP to separate individual oscillatory components within CA1. (C) CSD power profiles averaged over theta cycles from s.p., s.r., and s.l.m electrodes. Notice the mixture of three gamma types (SG, 30–50 Hz; MG, 60–100 Hz; and fast gamma, >100 Hz) at the s.p. electrode. (D) Normalized LFP power from the s.p. electrode averaged across theta cycles for running (speed ≥ 2 cm/s) and stillness (speed < 2 cm/s). (E) Example: 1.5-s LFP obtained from the s.p. electrode (white) and its time-frequency representation obtained by wavelet transform. Each frequency band was normalized separately by dividing signal power with signal variance. Notice the presence of mixed states when SG and MG oscillations are present in a single theta cycle. Individual theta cycles are marked by vertical lines. Oscillatory events detected as local maxima in time-frequency 2D space with peak power >2.5 SD are marked with red crosses. CA1, Cornu Ammonis; CSD, current source density; LFP, local field potential; MG, mid-frequency gamma; SG, slow gamma; s.l.m., stratum lacunosum moleculare; s.p., stratum pyramidale; s.r., stratum radiatum; SWR, sharp-wave ripple. Underlying data can be found here: [https://goo.gl/oHH22A]. (TIF) [file pbio.2003354.s003.tif]

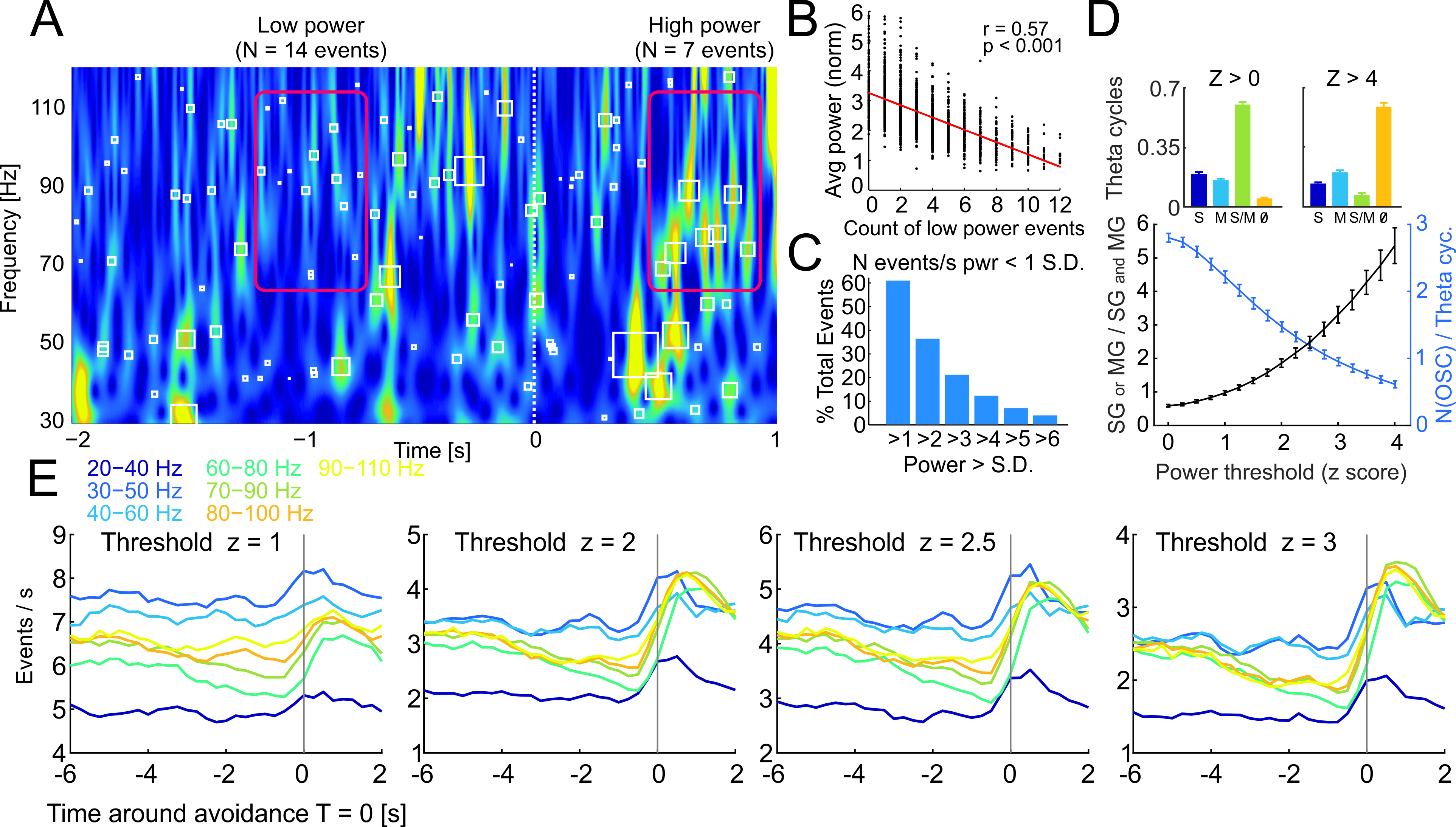

Supplement: S3 Fig — (A) Example time-frequency representation of LFP before and after avoidance. Power in the mid-frequency gamma range during stillness prior to avoidance is typically attenuated, leading to a higher number of detected low power events (N = 13, red rectangle around T = −1 s). Power in both slow and mid-frequency gamma ranges is typically increased during running away from the shock zone, leading to a higher number of detected high power events (N = 7, red rectangle around T = +0.75 s). (B) Average normalized power in a 1-s interval is negatively correlated with the number of low power events (z < 1) in the interval. (C) The proportion of detected events after applying different power thresholds. (D) Top: ratio of detected theta cycles with only S, M, S/M, and no detected oscillations (Ø) for power threshold z > 0 (left) and z > 4 (right). Bottom: relationship between the power threshold and the ratio of theta cycles with a single type of oscillation (slow or mid-frequency gamma) and the ratio of theta cycles with mixed oscillations (slow and mid-frequency gamma; black), plotted together with the number of supra-threshold oscillations per theta cycle (blue). (E) The average oscillation rates for 20-Hz wide bands covering the 20–110-Hz frequency range around avoidance onset (T = 0) for power thresholds z ≥ 1, 2, 2.5, and 3. Only the average profiles are included for clarity. LFP, local field potential; M, mid-frequency gamma; S, slow gamma; S/M, mixture of slow and mid-frequency gamma. Underlying data can be found here: [https://goo.gl/oHH22A]. (TIF) [file pbio.2003354.s004.tif]

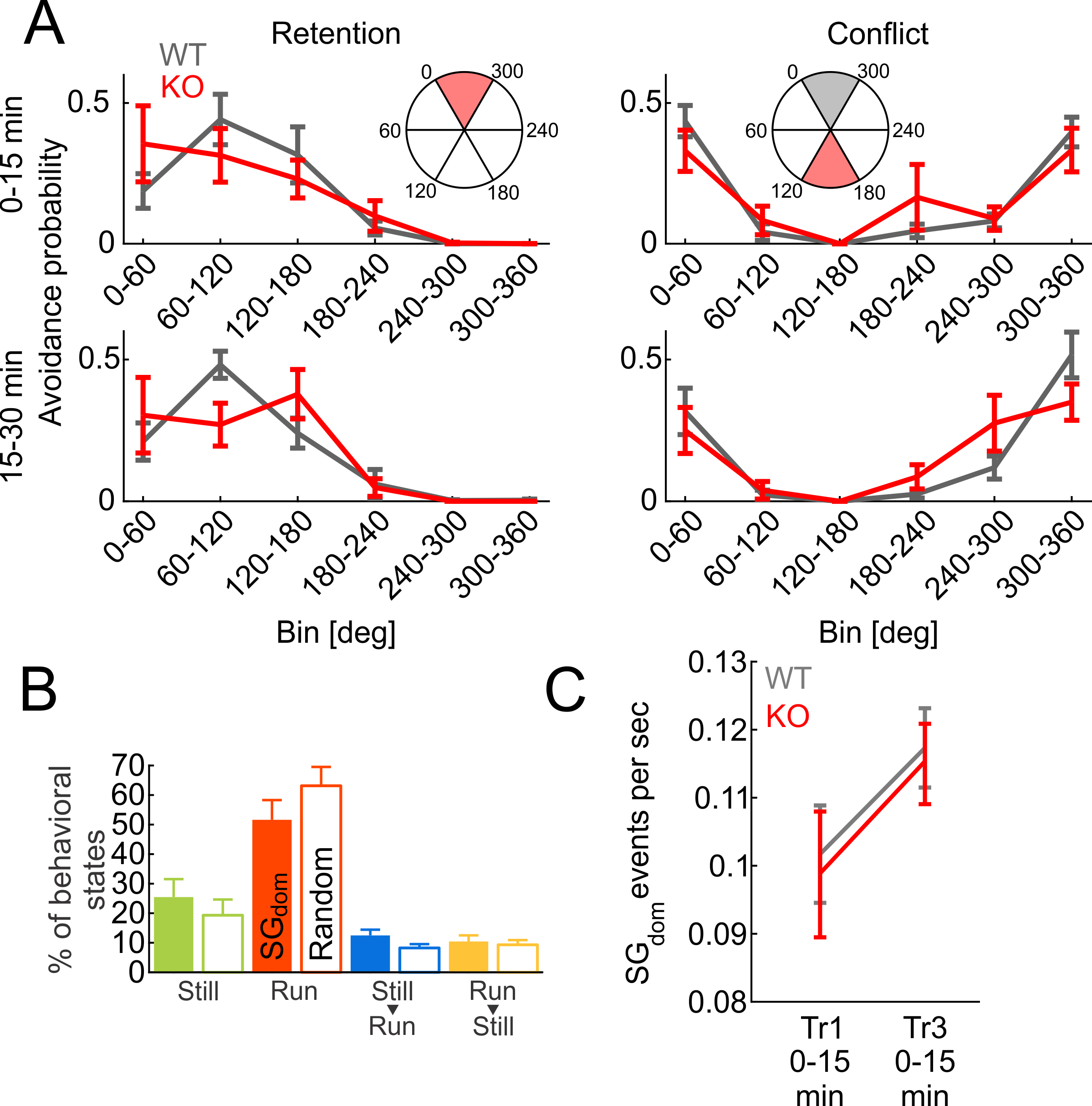

Supplement: S4 Fig — (A) Locations of avoidances across six 60° sectors during the first and second halves of the memory retention and conflict sessions. Avoidance profiles analyzed by two-way ANOVA with repeated measures; only the second half of the conflict session (15–30 minutes; S4A Fig bottom, right) shows a significant genotype × bin interaction (F5,8 = 4.56, p = 0.03), as Fmr1-KO animals display stronger avoidance of locations associated with the initial shock zone (0–60°, 300–360°). (B) Proportions of different behavioral events detected during SGdom events during pretraining sessions before ever experiencing shock (filled bars) compared to randomly selected events (empty bars; comparisons of SGdom to Random Still: χ12=6.5, p = 0.16; Run: χ12=8.3,p = 0.08; Still→Run: χ12=9.3, p = 0.05; Run→Still: χ12=0.004, p = 0.99). (C) Average SGdom rates across initial 15 min of first and last training sessions (two-way ANOVA with repeated measures genotype × trial: genotype: F1,13 = 0.30, p = 0.59; trial: F1,13 = 6.91, p = 0.02; genotype × trial: F1,13 = 0.04, p = 0.85). KO, knockout; SG, slow gamma. Underlying data can be found here: [https://goo.gl/oHH22A]. (TIF) [file pbio.2003354.s005.tif]

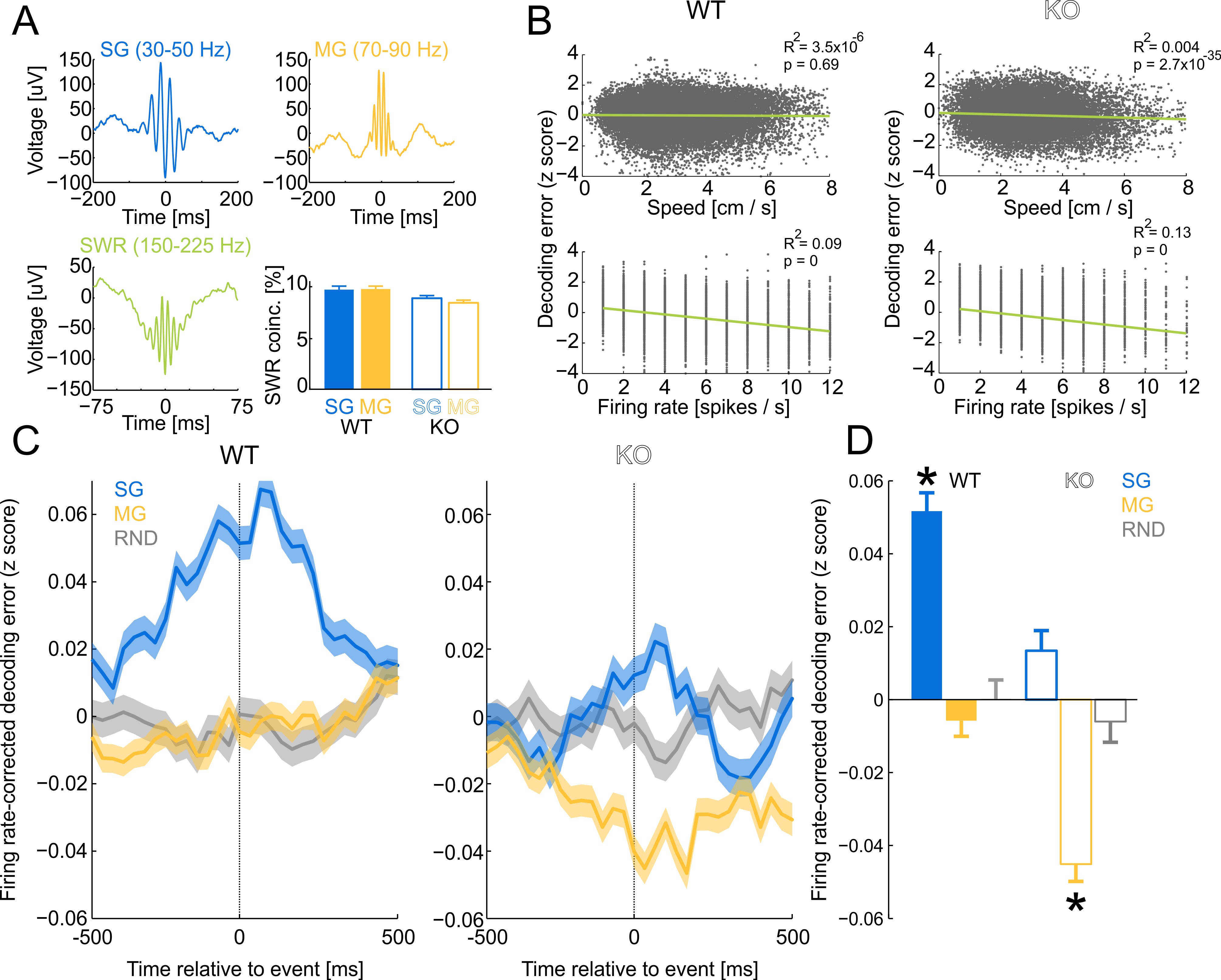

Supplement: S5 Fig — (A) Average voltage traces during isolated SG, MG, and SWR events. Percentage of (non-isolated) SG and MG events that coincide with SWR events. (B) Relationships between location decoding error (smaller error = more accurate) and running speed (top) and ensemble firing rate (bottom). (C) Bayesian decoding error during SG oscillations that are not accompanied by MG oscillations or SWRs (blue), MG oscillations that are not accompanied by SG oscillations or SWRs (yellow), and random events that are not accompanied by SWRs (gray) in WT and KO mice, corrected for firing-rate bias of the decoding. (D) Summary of decoding error during isolated SG and MG oscillations and random events. *p < 0.05 relative to random events. KO, knockout; MG, mid-frequency gamma; SG, slow gamma; SWR, sharp-wave ripple; WT, wild-type. Underlying data can be found here: [https://goo.gl/oHH22A]. (TIF) [file pbio.2003354.s006.tif]

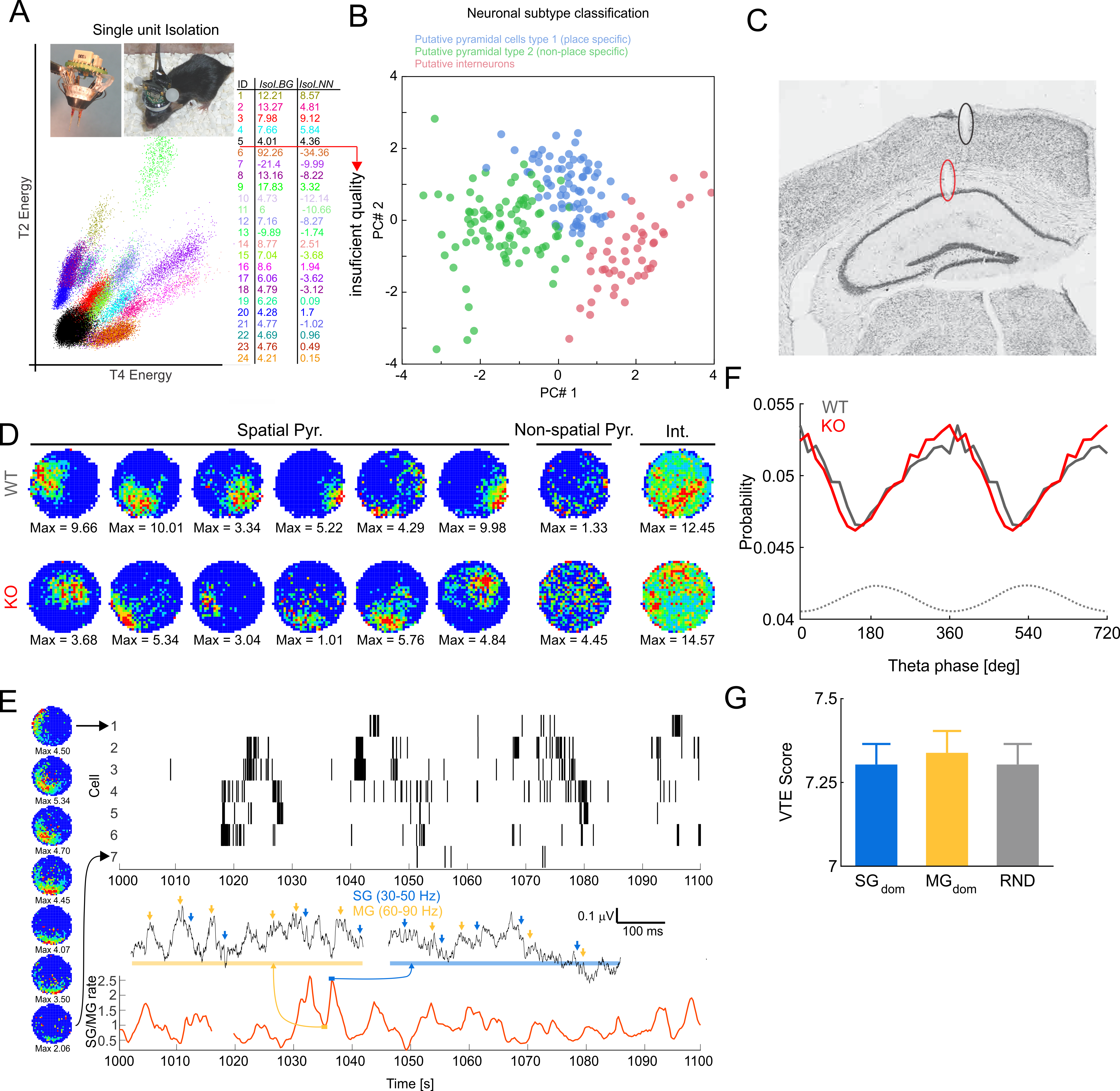

Supplement: S6 Fig — (A) Example of single-unit isolation and the Open Ephys microdrive (left inset) and an implanted mouse (right inset). The inset table lists isolated units with their corresponding IsoIBG and IsoINN values used for selecting units with sufficient isolation quality. Colors in the table correspond to clusters on the left. Only units with both quality measures >4.0 were analyzed further. (B) Neuronal subtype classification into three subtypes representing putative pyramidal cells with spatial specificity (type 1; blue), putative pyramidal cells without spatial specificity (type 2; green), and putative interneurons (red). Each dot represents a single well-isolated unit. Plot in 2D principal component space computed from the original 7D feature space that describes each unit. These features are the largest spike’s width, the unit’s firing rate, proportion of active pixels, firing-rate map coherence, firing-rate map information content, peak ISI, and proportion of spikes in a burst (≤10 ms ISI). (C) Histology showing electrode placement in CA1. Red and black ellipses mark tip of tetrode and point of entering cortex, respectively. (D) Example spatial putative pyramidal cells, nonspatial putative pyramidal cells, and putative interneurons from example wild-type (top row) and Fmr1-KO (bottom row) animals. (E) A seven-cell ensemble of spatially tuned putative pyramidal cells with their corresponding firing-rate maps (left) and raster plots of firing (top, right) during 100 s. The corresponding SG/MG ratio is shown in red (bottom, right), with LFP waveforms around the SG/MG maxima and minima, with identified SG (blue arrows) and MG (yellow arrows) oscillatory events. (F) Theta (8-Hz) phase preference of spatially tuned putative pyramidal cell discharge for wild-type (gray) and Fmr1-KO (red) mice. (G) Vicarious trial-and-error score computed for SGdom, MGdom, and random events. CA1, Cornu Ammonis 1; ISI, inter-spike interval; KO, knockout; MG, mid-frequency gamma; SG, slow gamma [file pbio.2003354.s007.tif]

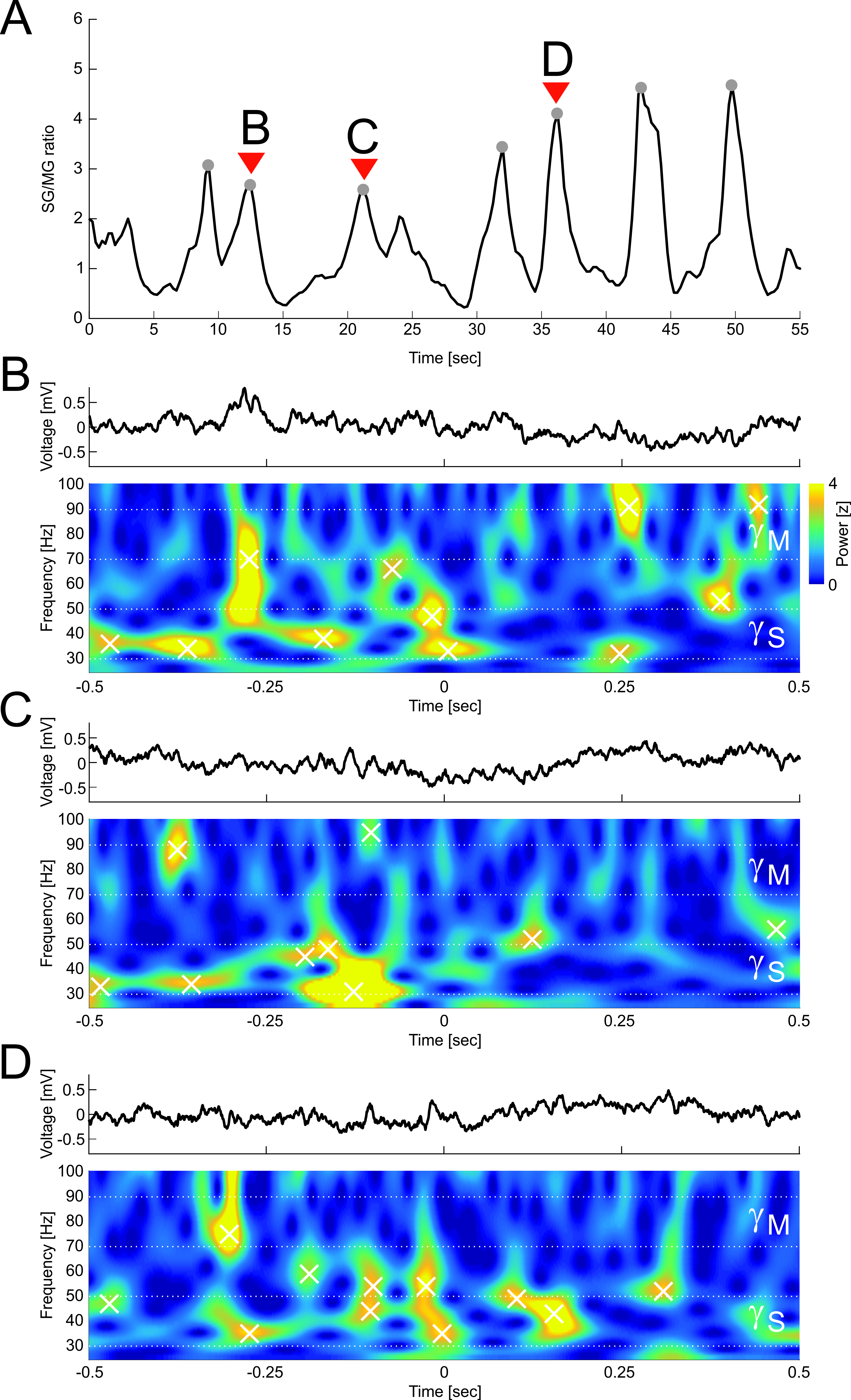

Supplement: S7 Fig — (A) SG/MG ratio computed over a 55-s-long time period, with identified SGdom peaks (gray circles) and a selected subset of SGdom peaks used for LFP and wavelet spectrum extraction in the examples below (red circles). (B–D) LFP (top) and wavelet spectrum (bottom) of 1-s-long segments centered on SGdom peaks. Power peaks with z-score power >2.5 were marked by a white cross. LFP, local field potential, MG, mid-frequency gamma; SG, slow gamma. Underlying data can be found here: [https://goo.gl/oHH22A]. (TIF) [file pbio.2003354.s008.tif]
