# Supplementary material for: Control of recollection by slow gamma dominating mid-frequency gamma in hippocampus CA1
Source: PLoS Biol. 2018 Jan 18;16(1):e2003354. doi: 10.1371/journal.pbio.2003354 (PMC5790293; doi:10.1371/journal.pbio.2003354)
Supplement: S1 Text — (DOCX) [file pbio.2003354.s009.docx]

**Basic LFP properties during active avoidance­**

Local field potentials analyzed during active avoidance training when mice were still (speed < 2 cm/s) and running (speed ≥ 2 cm/s; Fig S1A, B) both expressed peaks in the theta and gamma bands. Power was greater during running than stillness in the 5-10 Hz theta band (t_1,8_ = 4.91, p = 0.001), 30-50 Hz slow gamma band (t_1,8_ = 2.45, p = 0.04) as well as the 70-90 Hz mid-frequency gamma band (t_1,8_ = 6.38, p = 0.0002).

We detected sharp-wave associated ripple events [1-4] using a published algorithm [5]. The rate of ripple events were generally low [6] and did not differ during overall stillness or during the stillness that preceded subsequent active avoidance movements (Fig S1C, F_1,28_ = 0.34, p = 0.7).

during 5-s long windows centered around the current time (vertical line on the left). The video uses the same data that are presented in Fig 2 and Fig S7.

REFERENCES

1. Carr MF, Jadhav SP, Frank LM. Hippocampal replay in the awake state: a potential substrate for memory consolidation and retrieval. Nat Neurosci. 2011;14(2):147-53. Epub 2011/01/29. doi: nn.2732 [pii]

10.1038/nn.2732. PubMed PMID: 21270783; PubMed Central PMCID: PMC3215304.

2. Csicsvari J, Hirase H, Mamiya A, Buzsaki G. Ensemble patterns of hippocampal CA3-CA1 neurons during sharp wave-associated population events. Neuron. 2000;28(2):585-94. Epub 2001/01/06. doi: S0896-6273(00)00135-5 [pii]. PubMed PMID: 11144366.

3. Jackson JC, Johnson A, Redish AD. Hippocampal sharp waves and reactivation during awake states depend on repeated sequential experience. J Neurosci. 2006;26(48):12415-26. Epub 2006/12/01. doi: 26/48/12415 [pii]

10.1523/JNEUROSCI.4118-06.2006. PubMed PMID: 17135403.

4. O'Neill J, Senior T, Csicsvari J. Place-selective firing of CA1 pyramidal cells during sharp wave/ripple network patterns in exploratory behavior. Neuron. 2006;49(1):143-55. Epub 2006/01/03. doi: S0896-6273(05)00961-X [pii]

10.1016/j.neuron.2005.10.037. PubMed PMID: 16387646.

5. Eschenko O, Ramadan W, Molle M, Born J, Sara SJ. Sustained increase in hippocampal sharp-wave ripple activity during slow-wave sleep after learning. Learn Mem. 2008;15(4):222-8. doi: 10.1101/lm.726008. PubMed PMID: 18385477; PubMed Central PMCID: PMCPMC2327264.

6. Kay K, Sosa M, Chung JE, Karlsson MP, Larkin MC, Frank LM. A hippocampal network for spatial coding during immobility and sleep. Nature. 2016;531:185-90. doi: 10.1038/nature17144.
