# Supplementary material for: Control of recollection by slow gamma dominating mid-frequency gamma in hippocampus CA1
Source: PLoS Biol. 2018 Jan 18;16(1):e2003354. doi: 10.1371/journal.pbio.2003354 (PMC5790293; doi:10.1371/journal.pbio.2003354)
Supplement: S2 Text — (DOCX) [file pbio.2003354.s010.docx]

**Mixtures of slow, mid-frequency and fast gamma oscillations in *stratum pyramidale* of mouse CA1­**

Concurrent local field potentials (LFPs), reflecting the synchronous synaptic activity at distinct input-defined anatomical locations within the mouse CA1 region of dorsal hippocampus were recorded using 32-ch linear silicon electrode array (Neuronexus, Ann Arbor, MI) in order to demonstrate the presence of three distinct gamma bands within *stratum pyramidale* (sp) as shown previously in rat [1, 2] and mouse [3, 4]. LFPs were first localized (Fig S2A) using sharp-wave associated ripples (SWR). The maximum amplitude of the ripple identified *stratum pyramidale*, the maximum amplitude of the sharp wave identified *stratum radiatum*, and the sharp wave reversal identified *stratum lacunosum moleculare*. CSD analysis [5] was then performed in order to separate individual oscillatory CSD components (Fig S2B). Theta (5-12 Hz) phase was then used to construct theta-averaged CSD power profiles (Fig S2C). While *stratum radiatum* and *stratum lacunosum moleculare* CSDs show the presence of two distinct oscillatory bands (slow gamma 30-60 Hz and mid-frequency gamma 60-120 Hz), *stratum pyramidale* shows three distinct gamma bands (slow gamma 30-60 Hz, mid-frequency gamma 60-90 Hz and fast gamma > 100 Hz). Similar results were obtained when power profiles were constructed using band specific LFPs from *stratum pyramidale* (Fig S2D) during running (speed ≥ 2cm/s) and stillness (speed < 2cm/s). Notably, slow gamma (30-60 Hz) oscillations were distinguished more clearly than in CSD power profiles, while fast gamma (> 100 Hz) was harder to distinguish in LFP power profiles. The ability to detect both slow and mid-frequency gamma oscillations in *stratum pyramidale* is important for two reasons. First, the SG/MG ratio associated with recollection can be sufficiently estimated from the electrodes located in the *stratum pyramidale*. Second, since hippocampal place cell recordings target *stratum pyramidale*, the LFPs recorded together with single units can be used to estimate the SG/MG ratio.

**Mixtures of slow and mid-frequency gamma oscillations during single theta cycles**

It was reported that in rat, slow and mid-frequency gamma oscillations tend to occur mutually exclusively during theta cycles [6], but we find instead that slow and mid-frequency gamma oscillations detected as discrete events (Fig S2E) are often mixed in mouse, as reported by others [4; see Fig S4]. It is possible however, that the common, non-exclusive appearance of slow and mid-frequency gamma oscillations in single theta cycles might be specific to mouse.

REFERENCES

1. Schomburg EW, Fernandez-Ruiz A, Mizuseki K, Berenyi A, Anastassiou CA, Koch C, et al. Theta phase segregation of input-specific gamma patterns in entorhinal-hippocampal networks. Neuron. 2014;84(2):470-85. doi: 10.1016/j.neuron.2014.08.051. PubMed PMID: 25263753; PubMed Central PMCID: PMC4253689.

2. Fernandez-Ruiz A, Herreras O. Identifying the synaptic origin of ongoing neuronal oscillations through spatial discrimination of electric fields. Frontiers in computational neuroscience. 2013;7:5. doi: 10.3389/fncom.2013.00005. PubMed PMID: 23408586; PubMed Central PMCID: PMC3569616.

3. Lasztoczi B, Klausberger T. Layer-Specific GABAergic Control of Distinct Gamma Oscillations in the CA1 Hippocampus. Neuron. 2014;81(5):1126-39. doi: 10.1016/j.neuron.2014.01.021. PubMed PMID: 24607232.

4. Lasztoczi B, Klausberger T. Hippocampal Place Cells Couple to Three Different Gamma Oscillations during Place Field Traversal. Neuron. 2016;91(1):34-40. doi: 10.1016/j.neuron.2016.05.036. PubMed PMID: 27387648.

5. Mitzdorf U. Current source-density method and application in cat cerebral cortex: investigation of evoked potentials and EEG phenomena. Physiol Rev. 1985;65(1):37-100. PubMed PMID: 3880898.

6. Colgin LL, Denninger T, Fyhn M, Hafting T, Bonnevie T, Jensen O, et al. Frequency of gamma oscillations routes flow of information in the hippocampus. Nature. 2009;462(7271):353-7. Epub 2009/11/20. doi: nature08573 [pii]

10.1038/nature08573. PubMed PMID: 19924214.
