# Supplementary material for: Control of recollection by slow gamma dominating mid-frequency gamma in hippocampus CA1
Source: PLoS Biol. 2018 Jan 18;16(1):e2003354. doi: 10.1371/journal.pbio.2003354 (PMC5790293; doi:10.1371/journal.pbio.2003354)
Supplement: S3 Text — (DOCX) [file pbio.2003354.s011.docx]

**Selecting the power threshold for oscillatory events**

From the nature of time-frequency representations, when power is reduced, the time-frequency profile loses dominant peaks and instead more low power peaks are detected (Fig S3A). This results in a negative correlation between the number of low power peaks in a given time interval and the total power of all events detected in the interval (Fig S3B). To avoid this bias, we identified an optimal power threshold so that the number of oscillations per unit of time is not influenced by increased counting of low power events. Setting the power threshold to above 2 S.D. from the overall mean band power selects about 35% of all detected events. Setting the power threshold above 3 S.D. selects about 20% of all detected events (Fig S3C). Fig S3D bottom shows the continuous relationship between power threshold applied to detected events in the 30-60 Hz slow and 60-90 Hz mid-frequency gamma range and the ratio of theta cycles classified as having a unique type of oscillation (slow or mid-frequency gamma) and theta cycles classified as having both slow and mid-frequency gamma oscillations. We only included periods when the mouse was running (speed ≥ 2 cm/s) and only included theta cycles with periods between 83 and 250 ms corresponding to frequencies between 4 and 12 Hz. Predictably, low power thresholds lead to high numbers of detected events but a low ratio of single types of event (slow or mid-frequency gamma) present in a given theta cycle (Fig S3D top, left; theta cycles only with slow gamma ‘S’, mid-frequency gamma ‘M’, mixture of slow and mid-frequency gamma ‘S/M’ and no detected oscillations ‘Ø’). In contrast, high power thresholds lead to a low number of detected events and a high prevalence of single gamma-type theta cycles (Fig S3D top, right).

Because of the continuous relationship between power threshold and the ability to classify a theta cycle as one with just slow or mid-frequency gamma, we used the ratio of slow to mid-frequency gamma events per time period throughout this report.

**Identifying a power threshold and frequency bands of interest for slow and mid-frequency gamma event rates.**

To investigate the apparent asymmetry in the relative declines of slow and mid-frequency gamma events that was observed in the wavelet spectrum prior to avoidance (Fig 1E in the main text), we first identified the specific frequency bands of interest (Fig S3E). We computed the rate of oscillatory events in 1-s long intervals that advanced by 250 ms. The event rates around the avoidance onset were averaged across 20 Hz-wide bands between 20 and 110 Hz and for power thresholds z ≥ 1, 2, 2.5 and 3 during the third training session. We then compared these profiles across all bands to look for similarities. The 20-40 Hz band followed similar patterns of activity as the other two slow gamma bands (30-50 Hz and 40-60 Hz), but its average event rates were generally low so we excluded this band from further analysis. The 30-50 Hz and 40-60 Hz slow gamma bands were distinct from bands in the mid-frequency gamma range (60-110 Hz) for power thresholds above z ≥ 1, because the attenuation of slow gamma was reduced prior to avoidance onset, in fact, there were peaks of increased slow gamma rates 2-4 s before the avoidance initiated and there was an earlier increase of the slow gamma event rate at avoidance onset compared to a delayed increase in the rate of mid-frequency gamma events. We selected the 30-50 Hz band to represent slow gamma (SG) oscillations and the 70-90 Hz band to define mid-frequency gamma (MG) oscillations. In all subsequent analyses we only included events that were 2.5 S.D. or greater than the average power in a given frequency band.
